# Molecular Targeting of HuR Oncoprotein Suppresses MITF and Induces Apoptosis in Melanoma Cells

**DOI:** 10.3390/cancers13020166

**Published:** 2021-01-06

**Authors:** Rebaz Ahmed, Ranganayaki Muralidharan, Akhil Srivastava, Sarah E. Johnston, Yan D. Zhao, Suhendan Ekmekcioglu, Anupama Munshi, Rajagopal Ramesh

**Affiliations:** 1 Department of Pathology, The University of Oklahoma Health Sciences Center, Oklahoma City, OK 73104, USA; rebaz-ahmed@ouhsc.edu (R.A.); mranga550@gmail.com (R.M.); akhil-srivastava@ouhsc.edu (A.S.); 2 Graduate Program in Biomedical Sciences, The University of Oklahoma Health Sciences Center, Oklahoma City, OK 73104, USA; 3 Stephenson Cancer Center, The University of Oklahoma Health Sciences Center, Oklahoma City, OK 73104, USA; daniel-zhao@ouhsc.edu (Y.D.Z.); anupama-munshi@ouhsc.edu (A.M.); 4 Department of Biostatistics and Epidemiology, The University of Oklahoma Health Sciences Center, Oklahoma City, OK 73104, USA; sarah-johnston@ouhsc.edu; 5 Department of Melanoma Medical Oncology, The University of Texas MD Anderson Cancer Center, Houston, TX 77030, USA; sekmekcioglu@mdanderson.org; 6 Department of Radiation Oncology, The University of Oklahoma Health Sciences Center, Oklahoma City, OK 73104, USA

**Keywords:** melanoma, HuR, MITF, metastases, siRNA, targeted therapy, nanoparticles

## Abstract

**Simple Summary:**

The human antigen R (HuR) protein regulates the expression of hundreds of proteins in a cell that support tumor growth, drug resistance, and metastases. HuR is overexpressed in several human cancers, including melanoma, and is a molecular target for cancer therapy. Our study objective, therefore, was to develop HuR-targeted therapy for melanoma. We identified that HuR regulates the microphthalmia-associated transcription factor (MITF) that has been implicated in both intrinsic and acquired drug resistance in melanoma and is a putative therapeutic target in melanoma. Using a gene therapeutic approach, we demonstrated silencing of HuR reduced MITF protein expression and inhibited the growth of melanoma cells but not normal melanocytes. However, combining HuR-targeted therapy with a small molecule MEK inhibitor suppressed MITF and produced a synergistic antitumor activity against melanoma cells. Our study results demonstrate that HuR is a promising target for melanoma treatment and offers new combinatorial treatment strategies for overriding MITF-mediated drug resistance.

**Abstract:**

Background: Treatment of metastatic melanoma possesses challenges due to drug resistance and metastases. Recent advances in targeted therapy and immunotherapy have shown clinical benefits in melanoma patients with increased survival. However, a subset of patients who initially respond to targeted therapy relapse and succumb to the disease. Therefore, efforts to identify new therapeutic targets are underway. Due to its role in stabilizing several oncoproteins’ mRNA, the human antigen R (HuR) has been shown as a promising molecular target for cancer therapy. However, little is known about its potential role in melanoma treatment. Methods: In this study, we tested the impact of siRNA-mediated gene silencing of HuR in human melanoma (MeWo, A375) and normal melanocyte cells in vitro. Cells were treated with HuR siRNA encapsulated in a lipid nanoparticle (NP) either alone or in combination with MEK inhibitor (U0126) and subjected to cell viability, cell-cycle, apoptosis, Western blotting, and cell migration and invasion assays. Cells that were untreated or treated with control siRNA-NP (C-NP) were included as controls. Results: HuR-NP treatment significantly reduced the expression of HuR and HuR-regulated oncoproteins, induced G1 cell cycle arrest, activated apoptosis signaling cascade, and mitigated melanoma cells’ aggressiveness while sparing normal melanocytes. Furthermore, we demonstrated that HuR-NP treatment significantly reduced the expression of the microphthalmia-associated transcription factor (MITF) in both MeWo and MITF-overexpressing MeWo cells (*p* < 0.05). Finally, combining HuR-NP with U0126 resulted in synergistic antitumor activity against MeWo cells (*p* < 0.01). Conclusion: HuR-NP exhibited antitumor activity in melanoma cells independent of their oncogenic B-RAF mutational status. Additionally, combinatorial therapy incorporating MEK inhibitor holds promise in overriding MITF-mediated drug resistance in melanoma.

## 1. Introduction

Melanoma is the most aggressive form of skin cancer and is associated with the highest mortality. An estimated 100,350 new melanoma cases (60,190 in men and 40,160 in women) are expected to be diagnosed in 2020 in the United States, and roughly 8% of these patients are expected to die of the disease [1]. The treatment option depends on the stage of melanoma. Primary tumors and patients with limited loco-regional metastases are resected. Advanced metastasized patients received systemic therapy with immunotherapy or targeted therapy. Targeted therapies for melanoma include small molecule inhibitors towards B-RAF^V600^ and MEK kinases (e.g., Vemurafenib, Dabrafenib, Trametinib, Cobimetinib and Binimetinib) that have been tested as monotherapy or combination treatments [2,3,4,5]. B-RAF^V600^ mutation, which is very common among melanoma patients, leads to constitutive activation of the mitogen-activated protein kinase (MAPK), leading to the proliferation and growth of melanoma cells [6,7]. Despite the clinical efficacy of B-RAF^V600^/MEK targeted inhibitors, a large subset of melanoma patients develop resistance due to the reactivation of other elements of the MAPK or the PI3K pathway and receptor tyrosine kinases (e.g., c-KIT) [8,9,10,11]. Therefore, a combination of B-RAF^V600^ and PI3K inhibitors were tested, but the clinical trial (NCT01616199, NCT01512251) showed no significant improvement of efficacy [12,13,14]. Although B-RAF/MEK combination therapy strategies have shown improvement in therapeutic outcomes, the development of acquired resistance is still a concern [15]. In addition to targeted therapy, immune therapy has gained attention as a promising therapeutic strategy for metastatic melanoma [16]. Recent advancements in immunotherapy for melanoma include the blockade of immune checkpoint proteins (ICPs), Programmed Death-1 (PD-1), Programmed Death-Ligand 1 (PD-L1), and cytotoxic T-lymphocyte-associated protein 4 (CTLA-4). Disrupting the interaction between the immune checkpoint proteins results in reactivating the immune system and eliminating tumor growth [17,18,19]. Despite the success of immunotherapy, obstacles still exist, including the inability to predict the treatment efficacy and development of resistance as well as the occurrence of immune-related adverse events (irAEs). Thus, challenges in effectively treating metastatic melanoma continue warranting testing of inhibitors against new therapeutic targets.

HuR is an RNA binding protein encoded by the embryonic lethal, abnormal vision like 1 (*ELAVL1*) gene located on chromosome 19p13.2 [20]. HuR protein binds the adenylate/uridylate (AU)- and U-rich elements (AREs) in the untranslated region (UTR) of mRNAs [21]. HuR stabilizes and shuttles mRNAs from the nucleus to the cytoplasm where they are translated. HuR has been shown to regulate the expression of many transcripts whose products are oncoproteins [22]. Therefore, overexpression of HuR in many cancer types, including oral, colorectal, gastric, lung, breast, ovarian, renal, and melanoma, has been identified and correlated with poor prognosis [23,24,25,26,27,28,29]. Consequently, HuR has gained attention as a target for cancer therapy. In recent years, our lab and others have demonstrated that the downregulation of HuR using small interfering RNA (siRNA) and small molecule inhibitors results in a global knockdown of proteins involved in cancer growth and metastases, consequently leading to suppression of tumor growth both in vitro and in vivo [30,31,32,33,34,35,36,37,38]. However, the role of targeting HuR and the effect of its inhibition on melanoma cell growth has not been reported.

In this study, we examined the inhibitory effect of human HuR-specific small interfering RNA (HuR-siRNA) encapsulated in a lipid nanoparticle (NP) on HuR in human melanoma cell lines in vitro. Our study showed that inhibiting HuR reduced HuR-regulated oncoproteins, including MITF, inhibited cell proliferation and cell cycle, diminished melanoma cell’s migration and invasion, and culminated in apoptotic cell death. Additionally, combinatorial therapy of HuR-NP and MEK1/2 inhibitor, U0126, produced synergistic anticancer activity compared to individual treatments. In conclusion, HuR-targeted monotherapy and combinatorial therapy with MEK1/2 inhibitor is a new approach for melanoma treatment.

## 2. Materials and Methods

### 2.1. Cell Lines

Human melanoma cell lines (MeWo, A375, SK-MEL3, and WM39) and primary human melanocytes were obtained from the American Type Culture Collection (ATCC, Rockville, MD, USA). The cell lines were authenticated to be of human origin by single tandem repeat assay (STR; IDEXX Bioresearch, Columbia, MO, USA). Melanoma cells were maintained in Dulbecco’s modified Eagle’s medium (DMEM), and Minimum Essential Medium (MEM) supplemented with 10% fetal bovine serum (FBS; Sigma Aldrich, St. Louis, MO, USA) and 1% penicillin/streptomycin. Melanocytes (ATCC) were maintained in Dermal Cell Basal Medium (DCBM) supplemented with Melanocyte Growth Kit (ATCC). The passage number of the melanoma cell lines used in this study ranged from 4 to 35. The passage number of melanocytes used in the study ranged from 3 to 8.

For generating MeWo-EGFP and MeWo-MITF cell line, MeWo cells seeded in six-well plates were stably transfected with 2 µg of pEGFP × 2-N1 (Empty vector), and pEGFP-N1-MITF-M plasmid (Addgene, Watertown, MA, USA) encapsulated in a cationic lipid nanoparticle (NP). At 72 h after transfection, neomycin (G418; 400 µg/mL) (Sigma Aldrich) was added to the cells and selected for fourteen days. The surviving cell colonies were trypsinized, expanded, and maintained in neomycin (100 µg/mL) and used for experiments presented in this study.

### 2.2. Synthesis and Preparation of siRNA Containing Nanoparticles

The lipid-based cationic DOTAP:cholesterol nanoparticles (NP) formulation that comprises cationic DOTAP:cholesterol was synthesized and characterized as previously described [30,32,39,40,41]. The NPs were used to encapsulate human HuR-specific siRNA (Dharmacon, Lafayette, CO, USA) and scrambled control siRNA as previously described [30,32] and labeled as HuR-NP and C-NP, respectively, and used in the present study.

### 2.3. Cell Viability Assay

Trypan blue exclusion assay method was used to test the cytotoxic effect of HuR-siRNA containing nanoparticles (HuR-NP) as previously described [30,32,41]. Briefly, cells (MeWo, 7 × 10^4^ cells/well; A375, 1 × 10^5^ cells/well; melanocytes, 2 × 10^5^ cells/well) were seeded in six-well plates and incubated overnight in a CO_2_ incubator at 37 °C. The following day, the tissue culture medium from the plates was replaced with an appropriate fresh serum-free culture medium and treated with nanoparticles (NP) containing HuR-specific siRNA (100 nM; HuR-NP) or scrambled siRNA (100 nM; C-NP). At six hours after HuR-NP and C-NP treatment, the culture medium was replaced with fresh 2% serum containing culture medium, and incubation continued. At 24 h and 48 h after treatment, the cells were harvested, and the number of viable cells in each treatment group was determined. Cells that did not receive any treatment served as control. The results were expressed as the percentage of viable cells over untreated control cells. The experiments were repeated at least three separate times for reproducibility and were analyzed using appropriate statistical methods.

For testing the inhibitory activity of HuR-NP treatment on MeWo-MITF cells, cells were seeded in six-well plates and treated with HuR-NP and C-NP (100 nM siRNA). All other experimental conditions, including end-point analysis, were similar to those described above.

For determining the combinatorial treatment effect of HuR-NP and MEK inhibitor (U0126), MeWo or MeWo-MITF cells (7 × 10^4^) seeded in six-well plates were treated with DMSO, C-NP, HuR-NP (100 nM siRNA), U0126 (20 µM; Cell Signaling Technology Inc., Beverly, MA, USA), C-NP plus U0126, and HuR-NP plus U0126. The treatment protocol was as follows: cells were first treated with C-NP and HuR-NP for six-hours in a serum-free culture medium. After six hours, the culture medium was replaced with a 2% FBS-containing culture medium, and U0126 was added. Cells were harvested at 24 h and 48 h post-treatment and analyzed for cell viability by trypan blue assay and molecular markers by Western blotting. The combination treatments’ synergistic inhibitory effect was analyzed using SynergyFinder 1.0 tool [42].

### 2.4. Quantitative (q) RT-PCR Assay

Total RNA from HuR-NP- and C-NP-treated cells and untreated control cells were isolated using Trizol reagent (Life Technologies, Grand Island, NY, USA), and the RNA quality was determined using Denovix DS11 spectrophotometer. Complementary DNA (cDNA) was synthesized from 1 µg RNA/sample using a QuantScript cDNA synthesis kit (Bio-Rad, Richmond, CA, USA). An amount of 3 µg of synthesized cDNA, quantified using Denovix DS11 spectrophotometer, was subjected to real-time quantitative reverse transcriptase (qRT)-PCR (Bio-Rad CFX96™TouchReal-Time PCR Detection System; Richmond, CA, USA) using the premix iQ SYBR green qRT-PCR kit (Bio-Rad) as previously described [30,41]. The oligonucleotide primers (Integrated DNA Technology, Coralville, IA, USA) and their sequences for the amplification of HuR, BCL-2, and 18S RNA are shown below.

Human HuR
Forward—5′ATGAAGACCACATGGCCGAAGACT 3′
Reverse—5′ TGTGGTCATGAGTCCTTCCACGAT 3′
Human BCL-2
Forward—5′ ATG TGT GTG GAG AGC GTC AA 3′
Reverse—5′ ACA GTT CCA CAA AGG CAT CC 3′
Human 18S
Forward—5′ tagtagggacgggcggtgtg 3′
Reverse—5′ cagccacccgagattgagca 3′

The PCR cycling parameters and all other experimental conditions followed have previously been described [41]. The cycle threshold (Ct) value assessed by qRT-PCR was calculated for the transcripts and was normalized to a housekeeping gene. The changes in mRNA expression levels were expressed as fold change relative to control. Each sample was run in triplicate. The experiments were repeated at least three times for reproducibility and subjected to statistical analysis.

### 2.5. Western Blotting

Total cell lysates prepared from treated and untreated cells were harvested at defined time-points and subjected to Western blotting analysis as previously described [30,32,41]. Primary antibodies were purchased from commercial vendors and used for detecting human HuR, p27, BCL-2, alpha-tubulin (Santa Cruz Biotechnology, Dallas, TX, USA), cyclin D1, cyclin E1, HIF1-α, MITF, VEGF-A, Caspase-9, and PARP (Cell Signaling Technology Inc., Beverly, MA, USA) and beta-actin (Sigma Aldrich, St. Louis, MO, USA). Protein bands were detected using appropriate horseradish peroxidase (HRP)-tagged secondary antibodies (Santa Cruz Biotechnology) and an enhanced chemiluminescence kit (Thermo Scientific, MA, USA). Protein expression levels were detected on a chemiluminescence imaging system (Syngene, Frederick, MD, USA), and the relative protein expression compared to beta-actin or alpha-tubulin was quantified using Gene Tools software (Syngene) as previously described [30,32,41]. Experiments were repeated at least three separate times for reproducibility, and the data were analyzed for statistical significance.

### 2.6. Cell Cycle Analysis

Melanoma cells (MeWo, 4 × 10^4^ cells/well, and A375, 6 × 10^4^ cells/well) and melanocytes (2 × 10^5^ cells/well) seeded in six-well plates were treated with HuR-NP and C-NP (100 nM siRNA). At 24 h and 48 h after treatment, the cells were harvested and washed with PBS, then fixed in absolute ice-cold ethanol for 30 min, followed by washing 2 times with PBS. Fixed cells were then resuspended in cell staining buffer (Invitrogen, Carlsbad, CA, USA) at a concentration of 2 × 10^5^ cells/mL, and 100 μL of the cell suspension was incubated with 1 μL of 100 μg/mL propidium iodide (PI) (Invitrogen, Carlsbad, CA, USA) for 15 min at room temperature. The final washing was performed with PBS to wash out the unbound PI. Cells were subsequently subjected to flow cytometric analysis, as previously described [30,31]. Cells not receiving any treatment served as untreated controls. Experiments were conducted at least three separate times for melanoma cells and two times for melanocytes and subjected to statistical analysis. The data represented are the averages of two experiments.

### 2.7. Annexin V Assay

Cells seeded in six-well plates (MeWo, 4 × 10^4^ cells/well, and A375, 6 × 10^4^ cells/well) were treated with HuR-NP and C-NP (100 nM siRNA). At 24 h and 48 h after treatment, cells were harvested and stained with annexin V conjugated to fluorescein isothiocyanate (FITC) and propidium iodide (PI) using a dead cell apoptosis kit (Invitrogen) according to the manufacturer’s protocol. Briefly, harvested cells were suspended in annexin V binding buffer at a concentration of 2 × 10^5^ cells/mL, and 100 μL of the cell suspension was incubated with 5 μL of annexin V FITC and 1 μL of 100 μg/mL PI for 15 min of at room temperature. At the end of the incubation, the cells were processed and subjected to flow cytometric analysis (FACSCalibur^TM^; BD Biosciences, Bedford, MA, USA). The number of viable (annexin V- and PI-negative), early apoptotic (annexin V-positive and PI-negative), and dead (annexin V- and PI-positive) cells was determined at excitation 488 nm and emission 530 nm using the Cell Quest software (FACSCalibur^TM^; BD Biosciences). Cisplatin (CDDP; 1 µg)-treated cells served as a positive control, and the cells receiving no treatment served as a negative control. The results were plotted as the percentage of cells undergoing apoptosis. Experiments were repeated three times and subjected to statistical analysis. 

### 2.8. Cell Migration and Invasion Assay

Cell migration was carried out as previously described [32,43]. Briefly, MeWo cells (3 × 10^4^) were seeded in the upper chamber of 8 μm transwell (BD Biosciences) and placed in individual wells of a six-well plate filled with 1 mL of serum-free RPMI-1640 medium. At 24 h after seeding, the cells in the upper chamber were transfected with HuR-NP and C-NP (100 nM siRNA) in a serum-free medium. After 6 h of transfection, the upper and lower chambers’ medium was replaced with 2% and 20% serum-containing media, respectively. Incubation was continued, and the experiment was terminated at 24 h and 48 h after transfection, at which time the inserts were removed and stained with crystal violet (Sigma Aldrich). The number of cells migrated to the lower chamber was counted using an inverted bright-field microscope, and the results were expressed as an average number of migrated cells per microscopic field and subjected to statistical analysis. The experiment was performed at least two separate times to ensure reproducibility.

Cell invasion assay was performed using Matrigel-coated 8 μm transwell chambers (BD Biosciences) as previously described. All of the experimental conditions and parameters followed were the same as described above for the cell migration assay. The number of invading cells to the lower chamber was counted using an inverted bright-field microscope, and the results were expressed as an average number of invaded cells per microscopic field and subjected to statistical analysis. The experiment was performed at least two separate times to ensure reproducibility.

### 2.9. Statistics

The study results were subjected to statistical analysis using SAS 9.4 statistical analysis software. Continuous outcome variables are summarized with means and standard deviations. One-way ANOVA model with Tukey’s adjusted *p*-values was used to assess all pairwise comparisons. Adjusted *p*-values less than 0.05 were considered statistically significant. 

## 3. Results

### 3.1. Genetic Knockdown of HuR Inhibited Cell Growth in Melanoma

**Figure 1:**
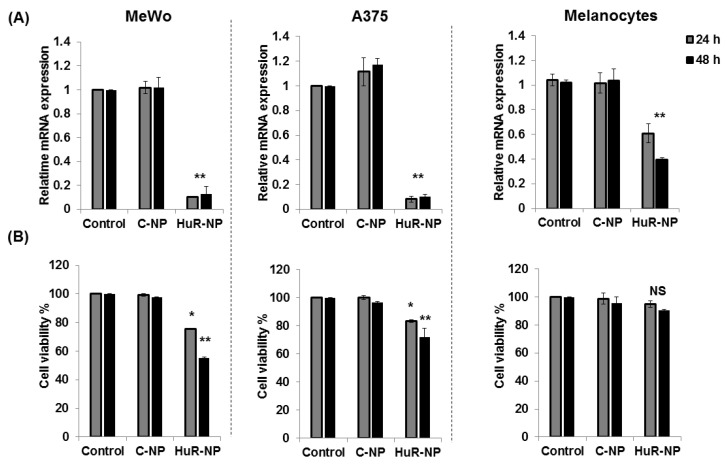
Effect of human antigen R (HuR)-nanoparticle (NP) treatment on HuR mRNA and cell viability. (**A**) HuR mRNA level and (**B**) cell viability was determined in human melanoma (MeWo, A375) cells and melanocytes treated with control siRNA-NP (C-NP) or HuR-NP (100 nM) for 24 h and 48 h. Untreated cells served as control. Error bar denotes SD; NS not significant; * *p* < 0.05; ** *p* < 0.01.

To validate HuR as a potential target for melanoma therapy, we first examined the expression pattern of HuR in a panel of human melanoma cell lines (A375.S2, WM39, SK-MEL-3, OCM-1, A375, WM1316A, OMM2.3, and MeWo) and primary normal human melanocytes. Our data revealed high HuR expression in all the melanoma cell lines examined and were independent of the B-RAF mutation status compared to HuR expression in melanocytes (Figure S1). For all of the remaining studies, we selected MeWo (B-RAF^wt^) and A375 (B-RAF^V600E^) as representative cell lines for melanoma and used melanocytes as control. Genetic knockdown of HuR using HuR-NP resulted in the attenuation of HuR mRNA in both MeWo and A375 cell lines at 24 h and 48 h (Figure 1A; *p* < 0.05). 

In melanocytes, HuR mRNA was also reduced upon HuR-NP treatment compared to C-NP and untreated control cells. However, the mRNA reduction in HuR-NP-treated melanocytes was markedly less compared to HuR-NP-treated melanoma cells. Next, we tested the inhibitory effect of HuR-NP treatment on the cell viability of melanoma cells and melanocytes. HuR-NP-treated MeWo cells showed approximately 25% and 45% inhibition at 24 h and 48 h, respectively, compared to C-NP-treated and untreated control cells (Figure 1B; *p* < 0.05). Similarly, HuR-NP treatment of A375 reduced cell viability up to 17% and 28% at 24 h and 48 h, respectively, compared to C-NP and untreated control (*p* < 0.05). To ensure the efficacy of HuR-NP is not limited to two melanoma (MeWo and A375) cell lines, cell viability was also evaluated in HuR-NP-treated SK-MEL-3 and WM39 melanoma cell lines. As shown in Figure S2, HuR-NP treatment significantly reduced SK-MEL-3 and WM39 cell viability at both 24 h and 48 h compared to C-NP-treated and untreated control cells. On the other hand, the treatment of melanocytes with HuR-NP exhibited a 6% and 10% reduction in cell viability at 24 h and 48 h, respectively, compared to C-NP and untreated controls (Figure 1B; *p* > 0.05). These results indicated that HuR-NP treatment, albeit reducing HuR mRNA in both melanoma cells and melanocytes, exerted selective and greater inhibitory activity on melanoma cell growth than in melanocytes.

### 3.2. Genetic Knockdown of HuR Using HuR-NP Reduced the Expression of HuR and HuR-Regulated Oncoproteins in Melanoma Cell Lines but Not in Melanocytes

**Figure 2:**
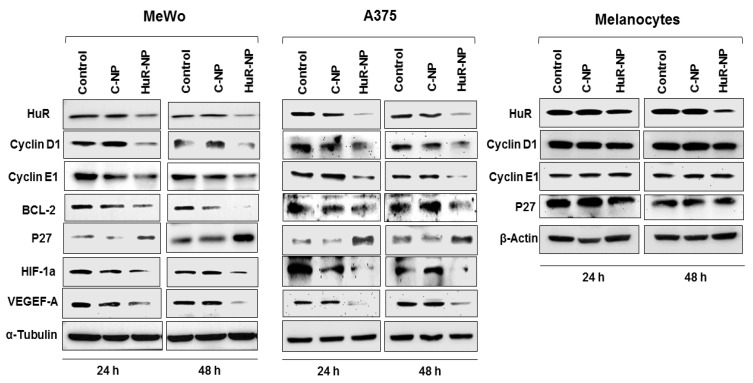
HuR-NP downregulates the expression of HuR and HuR-regulated proteins. Expression of HuR and HuR-regulated proteins in melanoma cell lines (MeWo, A375) and melanocytes at 24 h and 48 h after treatment with either C-NP or HuR-NP. Untreated cells served as control. β-actin and α-tubulin were used as loading controls.

HuR is known to stabilize many transcripts whose products are oncoproteins. To determine the role of HuR-NP treatment on the HuR target-oncoproteins, total cell lysates prepared from melanoma (MeWo, A375) cells and melanocytes receiving various treatments were examined by Western blot analysis. HuR-NP treatment significantly diminished protein expression of HuR, Cyclin D1, Cyclin E1, BCL-2, HIF1-α, VEGEF-A, and an increased expression of p27 at both 24 h and 48 h in melanoma cell lines (Figure 2 and Figure S3; *p* < 0.05). A similar observation of HuR silencing, resulting in BCL-2 reduction, was observed in SK-MEL-3 and WM39 melanoma cell lines (Figure S2).

We did not analyze for additional protein markers in SK-MEL-3 and WM39 melanoma cells that were examined in MeWo and A375 cells. Intriguingly, although HuR-NP treatment reduced HuR protein in melanocytes, no significant reduction in the expression of Cyclin E1, Cyclin D1, and p27 was observed (Figure 2 and Figure S3). The molecular mechanism for the observed differences in HuR-mediated downregulation of its targets between melanoma cell lines and melanocytes is unclear and warrants further investigation. Together, these results indicated that melanoma cell lines independent of the B-RAF mutational status were highly sensitive and responded to HuR-NP treatment compared to melanocytes.

### 3.3. Genetic Knockdown of HuR Induced G1 Cell Cycle Arrest in Melanoma

**Figure 3:**
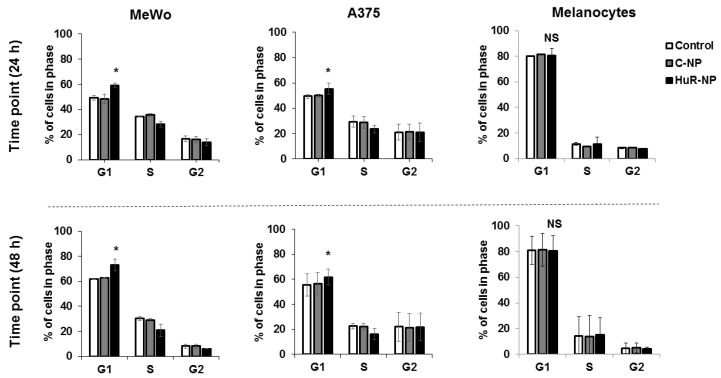
HuR-NP treatment induced G1 phase cell-cycle arrest in melanoma cell lines but not in melanocytes. Cell cycle analysis of melanoma cell lines (MeWo, A375) and melanocytes, at 24 h and 48 h after treatment with either C-NP or HuR-NP. Untreated cells served as control. Error bar denotes SD; NS not significant; * *p* < 0.05.

Since HuR knockdown reduced cyclin D1 and cyclin E1 and concomitantly increased the expression of p27, we evaluated the cell cycle profile of melanoma (MeWo and A375) and melanocytes with and without the HuR-NP exposure. Our results demonstrated that HuR-NP treatment significantly enriched the MeWo and A375 melanoma cells in the G1 phase of the cell cycle compared to C-NP-treated and untreated control cells (Figure 3). In HuR-NP-treated MeWo cells, an increase of 9% and 11% of cells in the G1 phase was observed at 24 and 48 h, respectively, compared to untreated and C-NP-treated cells (*p* < 0.05). In A375, about 5% and 8% increase in the number of cells in the G1 phase at 24 h and 48 h, respectively, was observed upon HuR-NP treatment compared to untreated and C-NP-treated cells (*p* < 0.05). However, HuR-NP did not alter the cell cycle phases in melanocytes at 24 h and 48 h after treatment. Our results revealed that the HuR-NP treatment selectively induced a G1 phase cell-cycle arrest in melanoma cells but not in melanocytes and concurred with previous results reported for other solid tumors [30,31,44].

### 3.4. Genetic Knockdown of HuR in Melanoma Cells Activated the Apoptosis Cascade

**Figure 4:**
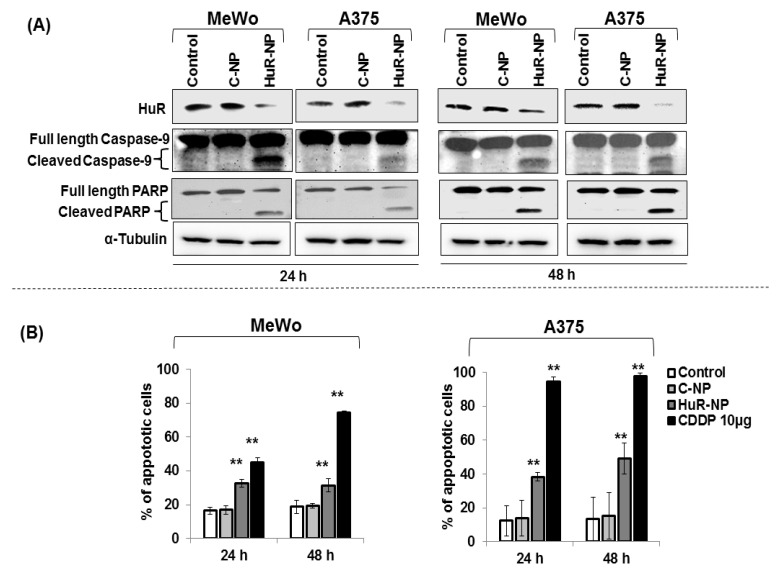
HuR-NP induced apoptosis in melanoma cell lines. Assessment of apoptosis in melanoma cell lines (MeWo, A375) at 24 h and 48 h after treatment with either C-NP or HuR-NP. Untreated cells served as control. (**A**), Western blotting showed caspase 9, and PARP cleavage was greater in HuR-NP-treated cells than in controls. (**B**), Annexin V staining was determined by flow cytometry. Cells treated with cisplatin (CDDP) served as a positive control for each cell line. Error bar denotes SD; ** *p* < 0.01.

To establish whether the reduction in anti-apoptotic protein BCL-2 in the HuR-NP-treated melanoma cells resulted in apoptosis, Western blot analysis for apoptotic proteins was performed. Cleavage of caspase-9, an indicator of activation of the caspase cascade, was greatly increased in both MeWo and A375 melanoma cells treated with HuR-NP compared to C-NP-treated and untreated control cells (Figure 4A and Figure S4). Accompanied by caspase 9 activation was the cleavage of its substrate, PARP. No significant activation of caspase-9 and PARP cleavage was observed in melanocytes following treatment with HuR-NP compared to HuR-NP-treated MeWo cells (Figure S5A,B).

Correlating with caspase activation in the melanoma cells was a significant increase in annexin-V-positive staining in HuR-NP-treated MeWo cells at 24 h (16% increase over controls) and 48 h (12% increase over controls), respectively, compared to C-NP-treated and untreated cells (Figure 4B; *p* < 0.05). In A375 cells, HuR-NP-treated cells showed a 25% and 33% increase in annexin-V-positive staining over controls at the two time-points tested (Figure 4B; *p* < 0.05). Cisplatin (CDDP; 10 µg)-treated cells were used as a positive control in the annexin V assay. The ability of HuR-NP treatment inducing apoptosis in melanoma cells but not in melanocytes is in agreement with prior reports showing HuR inhibition in tumor cells but not in normal cells results in apoptotic cell death [30,31,44].

### 3.5. Genetic Knockdown of HuR Inhibited Melanoma Cell Migration and Invasion

**Figure 5:**
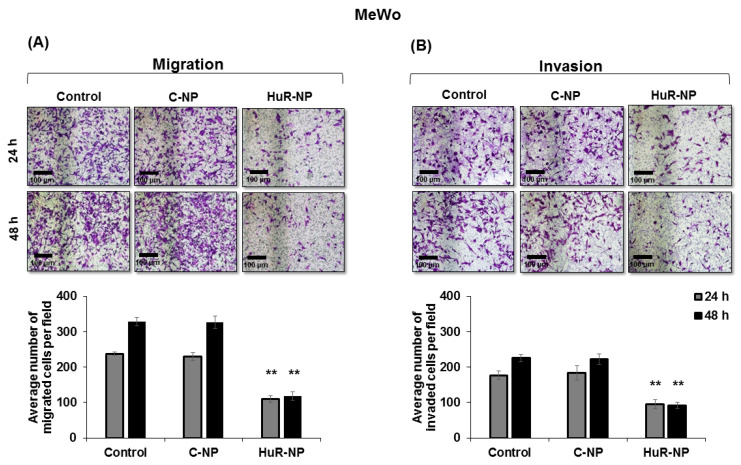
HuR-NP reduced melanoma cell migration and invasion. Representative images of crystal violet stained MeWo cells in (**A**), migration, and (**B**), invasion assay. Bar graphs represent the quantification of migrating and invading cells. Scale bar 100 µm. Error bar denotes SD; ** *p* < 0.01.

Migration and invasion are critical events in tumor metastases [45,46]. Previous studies have shown that HuR inhibition reduced tumor cell migration and invasion [30,31,32]. Therefore, we investigated the HuR-NP inhibitory effect on melanoma migration and invasion. HuR-NP treatment significantly reduced the number of migrated MeWo cells by 55% and 65% over C-NP-treated and untreated control cells at 24 h and 48 h, respectively (Figure 5A; *p* < 0.05). Similarly, testing the HuR-NP inhibitory effect on tumor cell invasion showed a significant reduction in the invasive ability of HuR-NP-treated MeWo cells by 46% and 60% over untreated and C-NP-treated cells at 24 h and 48 h, respectively (Figure 5B; *p* < 0.05).

### 3.6. HuR Inhibition Reduced MITF in Melanoma Cells

**Figure 6:**
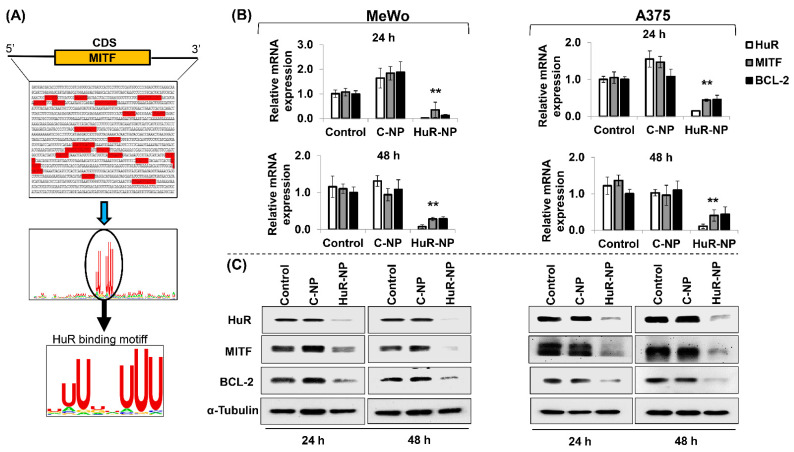
HuR-NP attenuates MITF mRNA and protein expression. (**A**), The MITF gene sequence, including promoter, untranslated regions (UTRs), and coding sequence (CDS), were scouted for HuR binding site. Multiple HuR binding sites were observed along the MITF gene. The conserved sequence search indicates HuR binding motif (5′NNUUNNUUU3′) conserved across the MITF gene sequence. RBPmap (http://rbpmap.technion.ac.il/) [51] and WebLogo (https://weblogo.berkeley.edu/) [52] available in the public domain as open resource tools were used for the analysis. The HuR-MITF binding was validated by (**B**), qRT-PCR, and (**C**), Western blot analysis in C-NP, and HuR-NP-treated MeWo and A375 melanoma cells. Untreated cells served as controls for each cell line. BCL-2 was assessed as positive control and downstream transcriptional target of MITF. 18S forward and reverse primers, and alpha α-Tubulin were used as internal controls in qRT-PCR and Western blotting, respectively. Error bar denotes SD; ** *p* < 0.01.

Studies have shown that MITF plays an important role in melanoma metastases and contributes to drug resistance [47,48,49,50]. Since HuR-NP treatment decreased cell invasion and migration, we investigated if HuR-NP treatment impacted MITF. Using gene sequence analysis, we analyzed for HuR-binding sites on MITF. As shown in Figure 6A, several HuR binding motifs were identified in the 3′ untranslated region (UTR) and coding sequence (CDS) of the MITF gene using the RBP map (https://rbmap.technion.ac.il) [51] and WebLogo (https://weblogo.berkeley.edu) [52]. Next, alterations in the expression levels of MITF mRNA and its downstream target, BCL-2, were assessed in HuR-NP-treated cells compared to untreated and C-NP-treated cells. A significant reduction in the mRNA expression of HuR, MITF, and BCL-2 was observed in HuR-NP-treated MeWo and A375 cells compared to their respective controls at the two time-points tested (Figure 6B; *p* < 0.05).

In accordance with the mRNA data, we observed a significant reduction in HuR, MITF, and BCL-2 protein expression levels in HuR-NP-treated MeWo and A375 cells at 24 h and 48 h compared to untreated and C-NP-treated cells (Figure 6C and Figure S6; *p* < 0.05). HuR-NP treatment of melanocytes showed no reduction in MITF compared to the untreated and C-NP-treated cells (Figure S7). To ensure consistency in our study results, MeWo cells receiving treatments identical to melanocytes were evaluated for MITF. HuR-NP treatment reduced MITF compared to untreated and C-NP-treated cells (Figure S7) and concurred with our data shown in Figure 6. Finally, immunostaining of a panel of human melanoma cell lines showed robust expression of MITF and HuR, albeit at varying levels for the two proteins in the cell lines (Figure S8). These results show that MITF is a molecular target of HuR and that inhibiting HuR concomitantly reduces MITF and its downstream BCL-2 expression. Furthermore, the MITF reduction in HuR-NP-treated cells likely contributes to the diminished cell migration and invasion observed in Figure 5.

### 3.7. HuR-NP Reduces U0126 Induced MITF in Melanoma Cells

Studies have shown that inhibiting the mitogen-activated protein kinase (MAPK) signaling pathway is beneficial in melanoma treatment [53,54,55]. Clinical studies testing inhibitors targeting MEK1/2, such as Trametinib alone and in combination with B-RAF inhibitor, Dabrafenib, have demonstrated clinical benefit in melanoma patients [56,57]. However, in the majority of the patients, the disease recurs and exhibits treatment resistance. Furthermore, high MITF expression in melanoma contributes to resistance towards MAPK inhibitors [58,59,60]. Based on these reports, we investigated whether incorporating HuR-NP with MEK1/2 inhibitor (U0126) will demonstrate improved efficacy.

Prior to conducting combinatorial efficacy studies, optimization studies testing different concentrations of U0126 (10 µM, 20 µM, and 30 µM) were performed and evaluated for cytotoxicity as well as MEK1/2 inhibition. Treatment of MeWo cells with U0126 resulted in a dose-dependent reduction in cell viability at 24 h and 48 h compared to DMSO-treated control cells (Figure S9; *p* < 0.05). Molecular analysis showed U0126 treatment while reducing phosphorylated (p)-MEK1/2^Ser217/221^ significantly increased MITF expression in a dose-dependent manner at 24 h and 48 h (Figure S9; *p* < 0.05).

**Figure 7:**
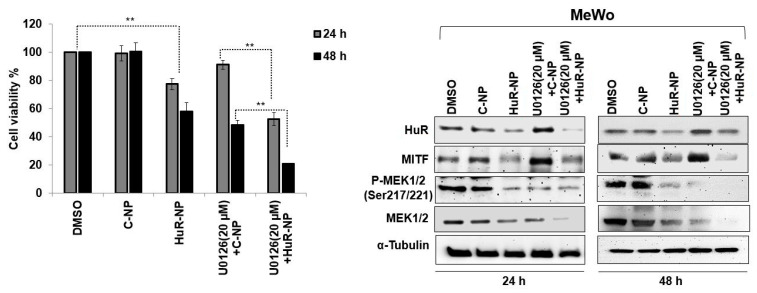
Cytotoxic effect of HuR-NP and U0126 combinatorial therapy in melanoma cell lines. Cells were treated with C-NP, HuR-NP, U0126, and a combination of C-NP or HuR-NP with U0126, and cell viability was assessed at 24 h and 48 h after treatment. DMSO-treated cells served as a control. HuR-NP plus U0126 treatment produced the greatest antitumor activity compared to all other treatment groups. Western blotting showed that HuR-NP plus U0126 combination treatment significantly reduced MITF, p-MEK1/2^Ser217/221^, and total MEK1/2 compared to all other treatment groups. Error bar denotes SD; ** *p* < 0.01.

Next, we investigated whether HuR-NP treatment could override U0126-treatment-induced MITF and exhibit increased efficacy. MeWo cells treated with HuR-NP showed significant inhibition of cell viability and marked reduction in MITF and p-MEK1/2^Ser217/221^ expression compared to DMSO- and C-NP-treated cells (Figure 7 and Figure S10; *p* < 0.05).

However, a synergistic effect in reducing cell viability and reduction in MITF and p-MEK1/2^Ser217/221^ expression was observed when HuR-NP was combined with U0126 compared to all other treatment groups (Figure 7 and Figure S10; *p* < 0.05). C-NP, in combination with U0126, produced an inhibitory effect akin to that observed with HuR-NP treatment alone but less than that with the combination treatment of HuR-NP and U0126. Another important observation was that the increased MITF expression observed in C-NP and U0126 combination treatment and attributed to U0126 was almost completely eliminated in HuR-NP and U0126 combination treatment, especially at 48 h after treatment (Figure 7 and Figure S10; *p* < 0.05). Our study results showed the HuR-NP suppressed U0126 induced MITF and produced enhanced antitumor activity in the melanoma cell line.

### 3.8. HuR-NP Suppresses the Cell Viability of MITF Overexpressing Melanoma Cell Line

Since our results showed HuR-NP treatment alone reduced MITF and U0126 induced MITF, we investigated whether HuR-NP can suppress MITF when overexpressed in melanoma cells analogous to that seen in melanoma patients. For this purpose, we first generated MITF- and GFP-overexpressing MeWo cell lines and labeled them as MeWo-MITF-M and MeWo-GFP, respectively. The two cell lines were characterized for cell viability, MITF, and MITF-regulated downstream markers (Figure S11). MITF overexpression increased MeWo-MITF-M cell number indicative of MITF’s ability to support cell proliferation compared to MeWo-GFP cells and parental MeWo cells (Figure S11). Furthermore, MITF overexpression greatly increased BCL-2 and HIF1-α expression in MeWo-MITF-M cells (Figure S11), both of which are downstream transcriptional targets of MITF [61,62].

**Figure 8:**
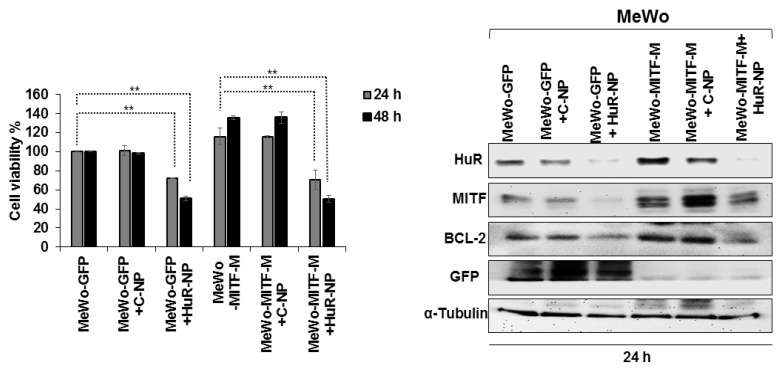
Cytotoxic effect of C-NP and HuR-NP treatment on MeWo-GFP and MeWo-MITF-M treated cells. HuR-NP significantly reduced the viability of both MeWo-GFP and MeWo-MITF-M cells at 24 h and 48 h compared to all other treatment groups. Western blotting showed HuR-NP reduced MITF, p-MEK1/2^Ser 217/221^ and total MEK1/2, and BCL-2 protein expression in both MeWo-GFP and MeWo-MITF-M cells. α-Tubulin was used as an internal loading control. Error bar denotes SD; ** *p* < 0.01.

Next, we tested HuR-NP’s ability to reduce the viability of MeWo-MITF-M cells and suppress MITF and its downstream targets. As shown in Figure 8, HuR-NP treatment significantly reduced MeWo-MITF-M cell viability compared to controls and was comparable to the HuR-NP inhibitory effect on MeWo-GFP cells at the two time-points tested (*p* < 0.05). Molecular analysis revealed HuR-NP unequivocally and effectively reduced MITF and BCL-2 in both MeWo-GFP and MeWo-MITF-M cells compared to their untreated and C-NP-treated control cells at 24 h after treatment (Figure 8 and Figure S12).

**Figure 9:**
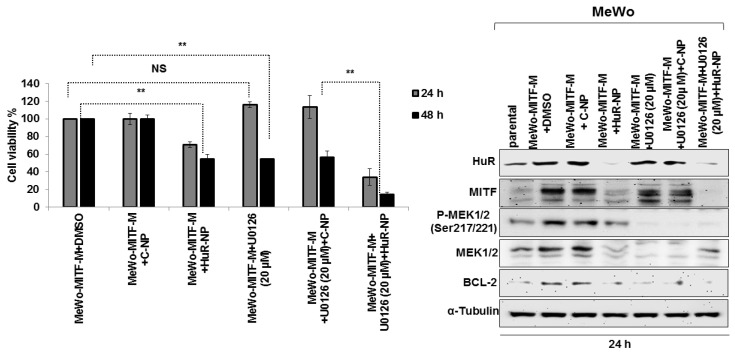
HuR-NP and U0126 combinatorial treatment produce enhanced antitumor activity in MeWo-MITF-M cells. Cells were treated with C-NP and HuR-NP in the presence or absence of U0126 and assessed for cell viability by trypan blue exclusion assay. DMSO-treated cells served as controls. Changes in molecular markers after treatment were examined by Western blotting assay. Combination treatment of HuR-NP and U0126 showed the greatest antitumor activity and maximum reduction in MITF. Reduction in p-MEK1/2^Ser 217/221,^ total MEK1/2, and BCL-2 observed in HuR-NP, and U0126 combination treatment was comparable to the reduction in these proteins in cells treated with C-NP plus U0126 and U0126 alone. Error bar denotes SD; ** *p* < 0.01.

Finally, we examined the combinatorial therapeutic efficacy of HuR-NP and U0126 on MeWo-MITF-M cells compared to individual treatments. HuR-NP and U0126 combination treatment produced a significant and greater inhibitory effect on MeWo-MITF-M cell viability with approximately 64% reduction at 24 h and 86% reduction at 48 h after treatment compared to all other treatment groups (Figure 9). The inhibitory effect on cell viability produced by U0126 treatment (46% inhibition) and C-NP and U0126 combination treatment (44% inhibition) was equivalent to the inhibitory effect produced by HuR-NP treatment alone (46% inhibition) at 48 h compared to DMSO-treated control cells (Figure 9). Molecular studies showed HuR-NP and U0126 combination treatment produced the greatest reduction in HuR, p-MEK1/2^Ser217/221^, BCL-2 protein expression, and most importantly, almost completely eliminated MITF expression in MeWo-MITF-M cells compared to all other treatment groups (Figure 9 and Figure S13). These results demonstrate the effectiveness of combining HuR-NP with MEK inhibitors to overriding the oncogenic effects of MITF and potentially mitigating MITF-mediated resistance in melanoma (Figure S13).

## 4. Discussion

Tremendous efforts to improve treatment outcomes for melanoma patients have met with limited success until the recent development of immunotherapy. However, limitations continue to exist with immunotherapy, and reports of resistance to immunotherapy and disease recurrence are emerging [63,64,65]. Enthusiasm for targeted therapies, especially towards B-RAF inhibitors (Vemurafenib, Dabrafenib) for melanoma treatment, continue to persist, and several combinatorial treatments incorporating MAPK inhibitors (Trametinib, Cobimetinib) are being pursued [66,67]. While co-targeting B-RAF and MEK1/2 have shown to improve treatment response and provide clinical benefit, the manifestation of treatment-related acquired resistance continues to evolve [49]. Therefore, efforts to develop and test improved therapies for melanoma are in pursuit.

This study established the benefit of targeting human antigen R, HuR, in melanoma. Studies have shown that HuR is a molecular target for therapy [22,27,68,69], and inhibiting HuR resulted in antitumor and antimetastatic activity [30,31]. No studies, however, have evaluated the effect of targeting HuR on melanoma cell growth and metastases. Activating B-RAF mutations are common and occur in approximately 50% of cutaneous melanomas [5,14]. However, our results clearly demonstrated that HuR overexpression occurred independent of B-RAF mutation status in melanoma cell lines compared to melanocytes. This observation encouraged us to investigate targeting HuR in melanoma. Therefore, we tested the genetic inhibition of HuR using HuR-specific siRNA containing nanoparticles (HuR-NP) in two melanoma cell lines differing in their B-RAF status (MeWo, B-RAF^wt^, and A375, B-RAF^V600E^). HuR-NP inhibited cell proliferation of both the cell lines independent of their B-RAF status. Molecular studies demonstrated that HuR-NP significantly reduced HuR mRNA and protein. Furthermore, HuR-NP attenuated HuR-regulated oncoproteins in both melanoma cell lines. HuR-NP-mediated inhibition led to a G1 phase cell cycle arrest that subsequently led to apoptotic cell death, as evidenced by the activation of the caspase cascade. While our results concurred with other studies that used different tumor models, it is also suggested that HuR-NP could be an attractive target for melanoma therapy independent of oncogenic B-RAF mutation status. Considering the role of HuR in metastases, we tested the impact of HuR-NP on an invasive MeWo melanoma cell line. Interestingly, HuR diminished the migratory and invasive ability of MeWo cells. Most importantly, our results showed that HuR-NP exerted selective cytotoxicity towards melanoma cells but not towards normal melanocytes, a feature that is preferred in having effective cancer treatment.

Next, we investigated whether the inhibitory effect on melanoma migration and invasion following HuR-NP treatment could partly be due to MITF. MITF has been reported to contribute to melanoma cell survival, cell migration and invasion, drug resistance, and metastases [49,70,71,72]. To our surprise, a marked reduction in MITF and MITF-regulated BCL-2 and HIF-1α proteins was observed upon silencing of HuR. The ability of HuR to suppress MITF at both the mRNA and protein levels is an interesting observation that is hitherto not reported. Based on this initial observation of HuR-NP reducing MITF, we conducted combinatorial studies using U0126, a MEK1/2 inhibitor, to emulate clinical studies conducted for treating melanoma patients. Use of MEK1/2 inhibitors such as Trametinib, either alone or in combination with B-RAF inhibitor, Dabrafenib, while showing initial clinical benefit, has failed to demonstrate long-term efficacy due to disease recurrence and drug resistance. Furthermore, the expression of MITF has been reported to play a role in the failure of MEK1/2-targeted therapy [50,73,74]. In fact, our in vitro results showed HuR-NP treatment markedly reduced MITF, MEK1/2, and p-MEK1/2^Ser217/221^ proteins, which concurred with previous study results that showed MEK1 mRNA as a HuR mRNA-target in intestinal epithelial cells [75]. Therefore, we speculated that incorporating U0126 into HuR-NP treatment will result in enhanced antitumor activity. We clearly and convincingly demonstrated that HuR-NP plus U0126 treatment in MeWo cells abolished MITF-induced MEK1/2 expression; suppressed MEK1/2 inhibitor-induced MITF; and overrides the anti-apoptotic effects of MITF in MITF overexpressing MeWo cells resulting in synergistic antitumor activity. Based on our study results, it is interesting to speculate that the combinatorial treatment of HuR-NP and U0126 will be very effective against melanoma cells that have developed acquired resistance to B-RAF/MEK inhibitors. However, the authors have not conducted this experiment as it was beyond the scope of the study but plan to test it in the future. Additionally, the study is limited to in vitro observations that need to be validated in in vivo melanoma models prior to advancing to clinical translation.

It is to be noted that while our study demonstrated the therapeutic benefits of HuR-targeted therapy for melanoma, several questions remain unanswered. For example, Slominski et al. reported melanogenesis regulated HIF-1α and HIF-1α-regulated target genes are involved in angiogenesis and cellular metabolism [76]. Similarly, the involvement of cyclic adenosine monophosphate (cAMP) and response binding protein element (CREB) in MITF activation and subsequent upregulation of melanogenesis-regulated gene coding for tyrosinase has been reported [77,78]. Since our study showed a link between HuR and MITF and that HuR is known to regulate HIF-1α and HIF-1α-regulated target genes such as VEGF, it will be of interest to investigate the role of HuR in melanogenesis and melanogenesis regulated tyrosinase in melanoma. A cross talk between MAPK and melanogenetic pathways are also strongly interconnected [79]. However, the exact role of HuR in regulating the MAPK pathway and its implications in melanogenesis is unknown. Finally, little to none is known about HuR expression and its role in uveal melanoma (UM). Interestingly, unlike cutaneous melanoma, UM is characterized by a very low mutational burden. In addition, B-RAF and N-RAS are rarely mutated, instead GNAQ or GNA11 mutations are frequently detected and known to activate the MAPK pathway also [80,81]. Hence, targeting HuR might be a treatment option in UM. However, laboratory studies on this are pending to date. Together, the results from the present study highlight the importance of targeting HuR in melanoma and concomitantly opening new avenues for investigating HuR in melanogenesis and UM.

## 5. Conclusions

We have established proof-of-concept and shown that targeting HuR represents a promising therapeutic option for melanoma treatment with or without oncogenic B-RAF mutation. Furthermore, inhibiting HuR offered the additional advantage of reducing MITF expression in melanoma cells, and combinatorial therapy targeting HuR and MEK1/2 produced synergistic antitumor activity. These results support additional combinatorial testing of HuR-targeted therapeutics in combination with B-RAF and MEK1/2 inhibitors for melanoma both in vitro and in vivo.

## Data Availability

Data is contained within the article or supplementary material.

## Supplementary Materials

The following are available online at https://www.mdpi.com/2072-6694/13/2/166/s1. Figure S1: The baseline expression level of HuR, MITF, p-MEK1/2^Ser217/221^, and total MEK1/2 along with B-RAF and N-RAS status in melanoma cell lines and melanocytes, Figure S2: Cytotoxicity activity of HuR-NP on melanoma cell lines, Figure S3: Semi-quantitative analysis of the protein expression detected by Western blot analysis in C-NP and HuR-NP-treated melanoma (MeWo, A375) cell lines and melanocytes at 24 h and 48 h after treatment, Figure S4: Semi-quantitative analysis of the cleaved Caspase-9 and PARP proteins detected by Western blot analysis in C-NP and HuR-NP-treated melanoma (MeWo, A375) cell lines at 24 h and 48 h after treatment, Figure S5: (A), HuR-NP induced apoptosis in MeWo cells but not in melanocytes. Gels of lighter and darker exposure for PARP and caspase 9 included. (B), Bar graphs represent the semi-quantitative analysis of the cleaved Caspase-9, PARP, and HuR proteins detected by Western blot analysis, Figure S6: Bar graphs represent the semi-quantitative analysis of the protein (HuR, MITF, BCL-2) expression in C-NP and HuR-NP-treated MeWo and A375 melanoma cells detected by Western blot analysis at 24 h and 48 h after treatment, Figure S7: HuR-NP treatment reduced MITF in melanoma (MeWo) cell line but not in melanocytes at 24 h and 48 h after treatment, Figure S8: Immunocytochemistry of HuR and MITF expression in a panel of human melanoma cell lines, Figure S9: Cytotoxic effect of U0126 on MeWo melanoma cell line, Figure S10: Bar graphs represent the semi-quantitative analysis of protein expression detected by Western blot analysis in MeWo melanoma cells treated with C-NP, HuR-NP, U0126, and a combination of C-NP or HuR-NP with U0126 at 24 h and 48 h after treatment, Figure S11: Cell viability of MeWo-MITF-M compared to MeWo-GFP and parental MeWo cells were assessed by trypan blue exclusion assay at 24 h and 48 h, Figure S12: Semi-quantitative analysis of the protein expression in C-NP- and HuR-NP-treated MeWo-GFP and MeWo-MITF-M cells detected by Western blot 24 h after treatment, Figure S13: Bar graphs represent the semi-quantitative analysis of the protein expression detected in MeWo-MITF-M cells treated with C-NP, HuR-NP, U0126 (20 µM), a combination of U0126 with C-NP or HuR-NP by Western blot 24 h post-treatment.
