# Supplementary material for: Molecular Targeting of HuR Oncoprotein Suppresses MITF and Induces Apoptosis in Melanoma Cells
Source: Cancers (Basel). 2021 Jan 6;13(2):166. doi: 10.3390/cancers13020166 (PMC7825065; doi:10.3390/cancers13020166)

Supplementary Materials

Molecular Targeting of HuR Oncoprotein Suppresses MITF and Induces Apoptosis in Melanoma Cells

Rebaz Ahmed, Ranganayaki Muralidharan, Akhil Srivastava, Sarah E. Johnston, Yan D. Zhao, Suhendan Ekmekcioglu, Anupama Munshi and Rajagopal Ramesh

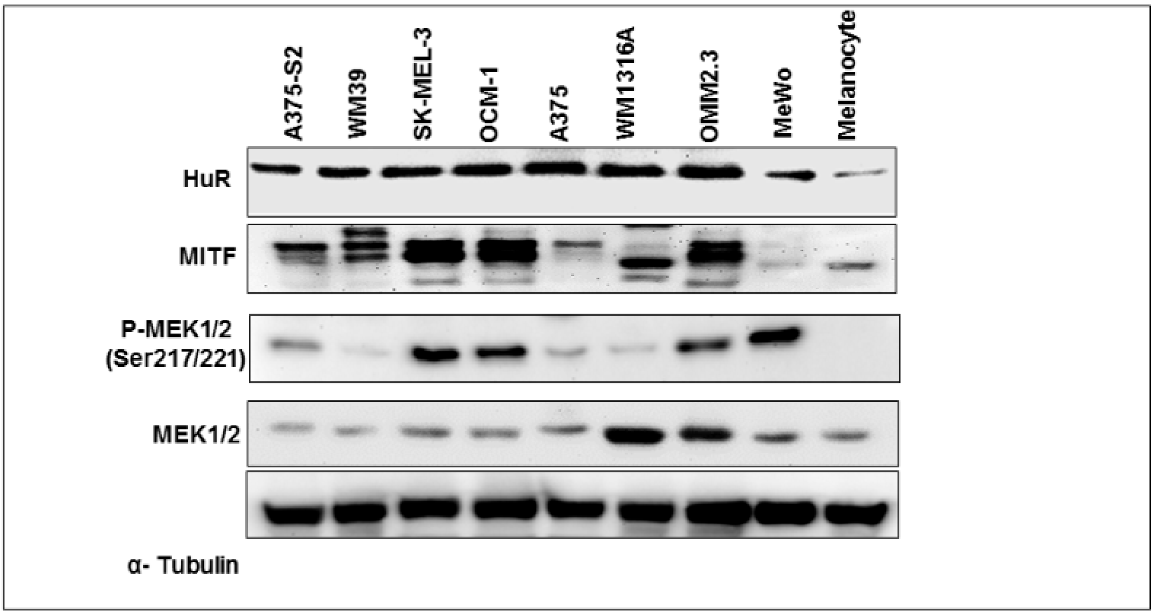

Figure S1. Baseline expression level of HuR, MITF, P-MEK1/2 (Ser217/221) and total MEK1/2 along with B-RAF and N-RAS status in melanoma cell lines and melanocytes.

Supplementary Table.1 B-RAF and N-RAS mutation status of melanoma cell lines and melanocyte. (WT: Wild type)

	A375-S2	WM39	SK-mel-3	OCM-1	A375	WM1361A	OCM2.3	MeWo	Melanocyte
B-RAF	V600E	V600E	V600E	V600E	V600E	WT	WT	WT	WT
N-RAS	WT	WT	WT	WT	WT	Q61R	Q61L	WT	WT

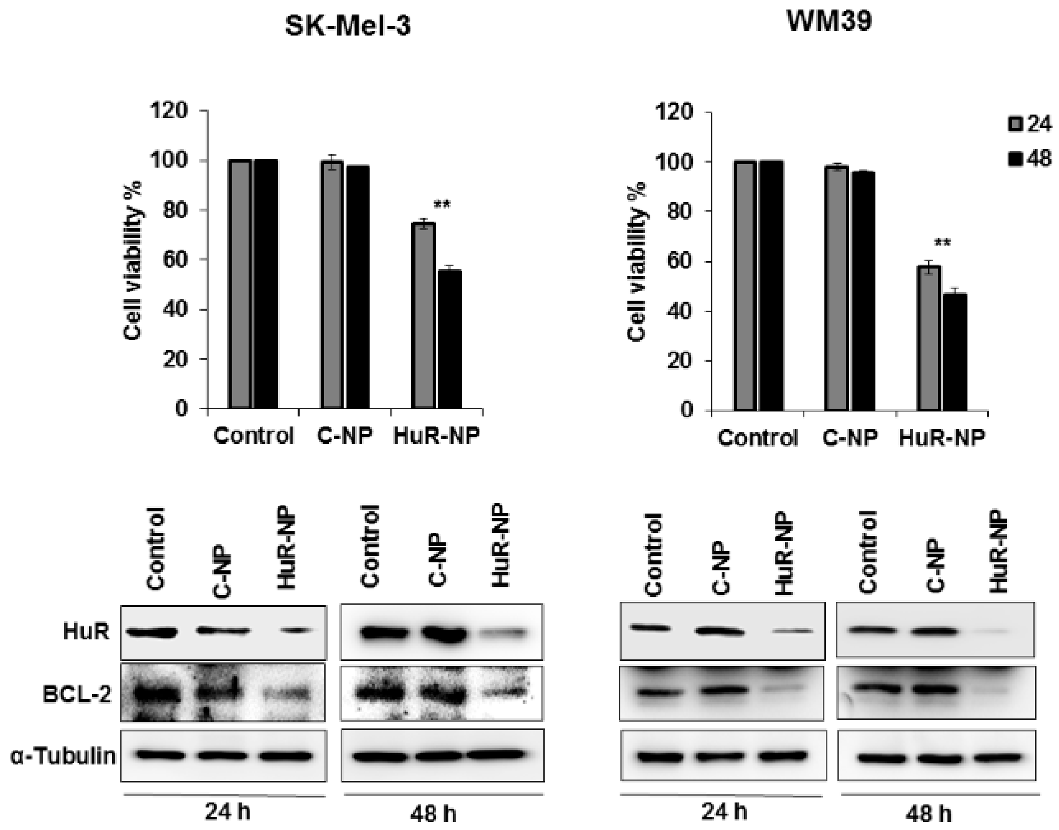

Figure S2. Cytotoxicity activity of HuR-NP on melanoma cell lines. Human SK-MEL3 and WM39 melanoma cell lines were treated with C-NP or HuR-NP and analyzed for cell viability by trypan-blue exclusion assay and for HuR and BCL-2 protein expression by western blotting. HuR-NP treatment significantly reduced the viability of SK-MEL3 and WM39 cells compared to C-NP treated and untreated control cells. Associated with reduction in cell viability was the marked decrease in HuR and BCL-2 protein expression in HuR-NP-treated cells. Error bar denotes SD; **p < 0.01.

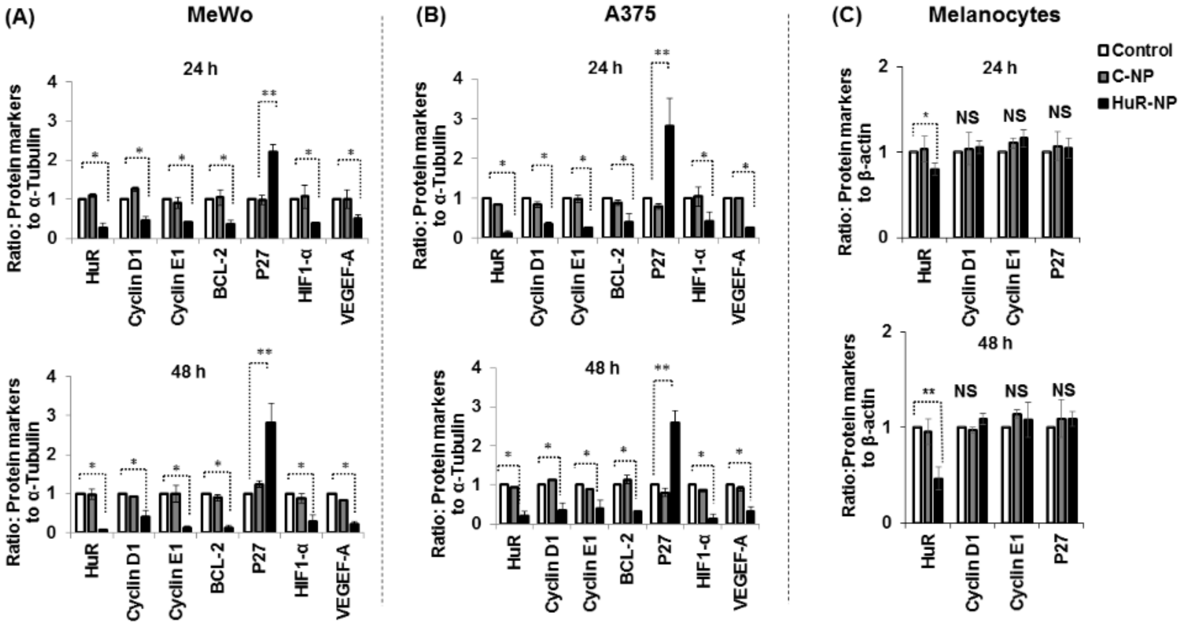

Figure S3. Semi-quantitative analysis of the protein expression detected by western blot analysis in C-NP and HuR-NP-treated melanoma (MeWo, A375) cell lines and melanocytes at 24 and 48 h after treatment. Untreated cells served as controls. Error bar denotes SD; NS not significant; *p < 0.05; **p < 0.01.

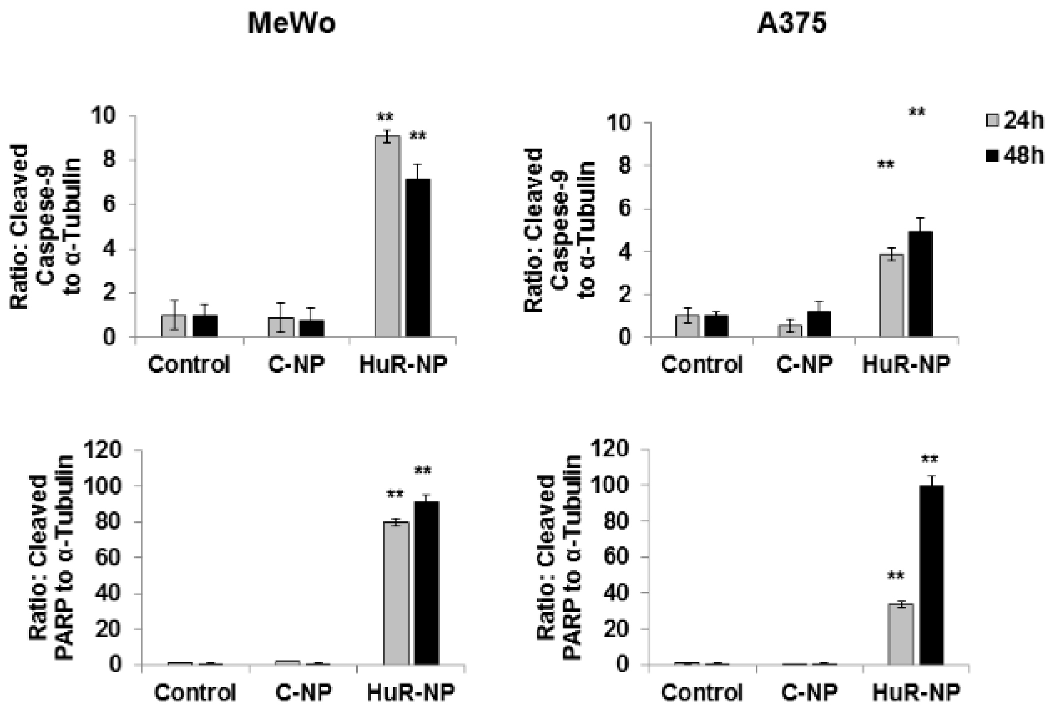

Figure S4. Semi-quantitative analysis of the cleaved Caspase-9 and PARP proteins detected by western blot analysis in C-NP and HuR-NP-treated melanoma (MeWo, A375) cell lines at 24 and 48 h after treatment. Untreated cells served as controls. Error bar denotes SD; **$p < 0.01$.

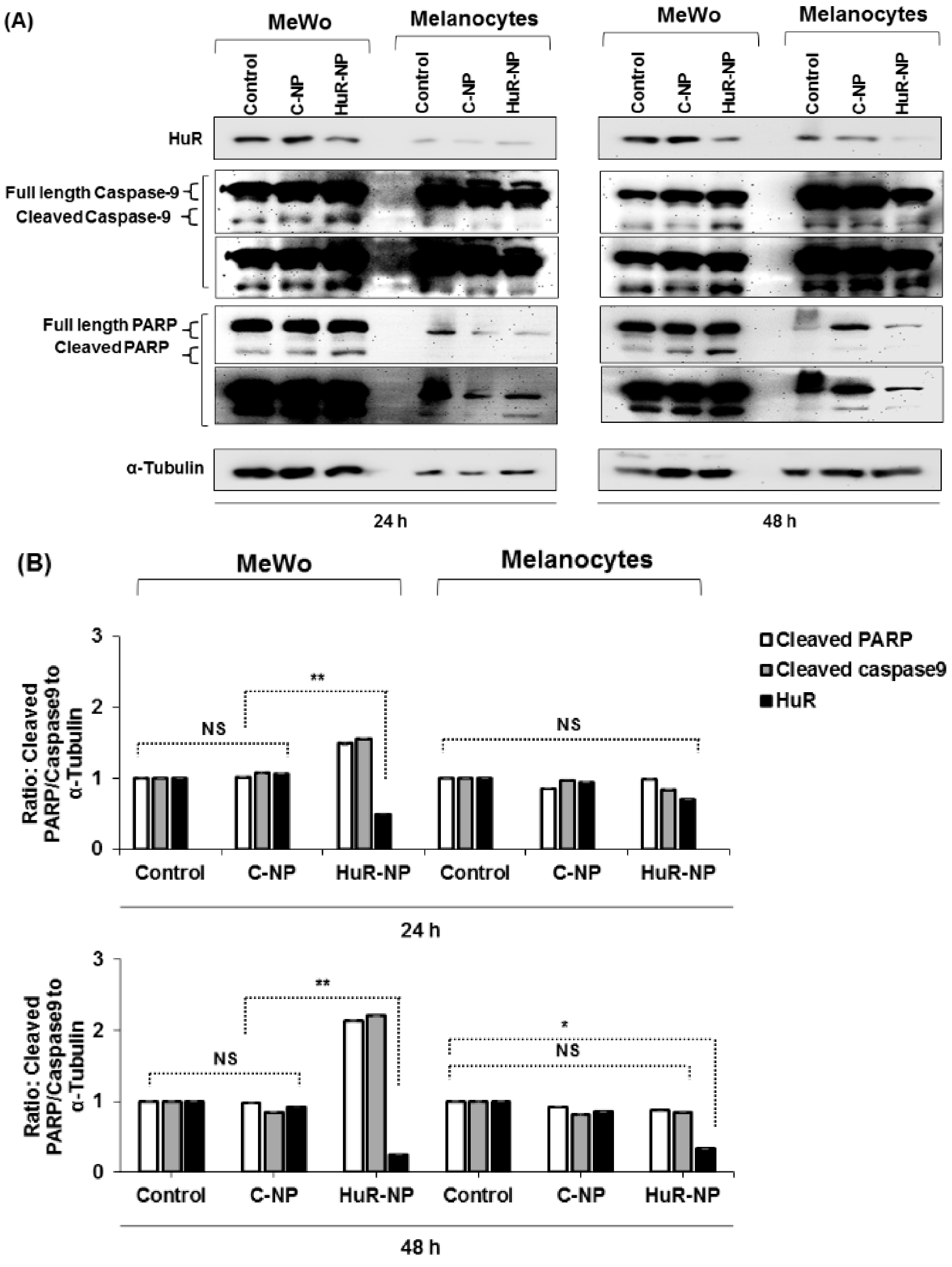

Figure S5. (A), HuR-NP-induced apoptosis in MeWo cells but not in melanocytes. Gels of lighter and darker exposure for PARP and caspase 9 included. (B), Bar graphs represent semi-quantitative analysis of the cleaved Caspase-9, PARP, and HuR proteins detected by western blot analysis. Error bar denotes SD; NS not significant; *p < 0.05; **p < 0.01.

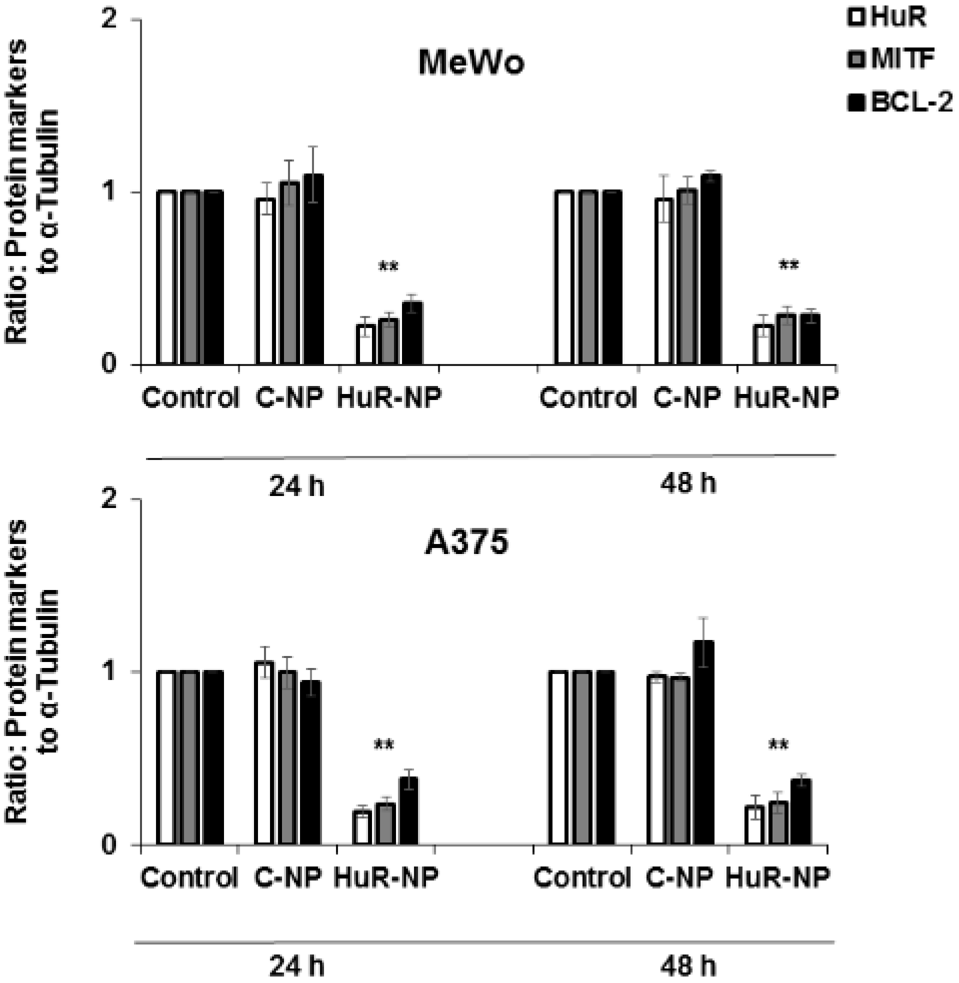

Figure S6. Bar graphs represent semi-quantitative analysis of the protein (HuR, MITF, BCL-2) expression in C-NP and HuR-NP treated MeWo and A375 melanoma cells detected by western blot analysis at 24 h and 48 h after treatment. Untreated cells served as control. Error bar denotes SD; NS not significant; **p < 0.01.

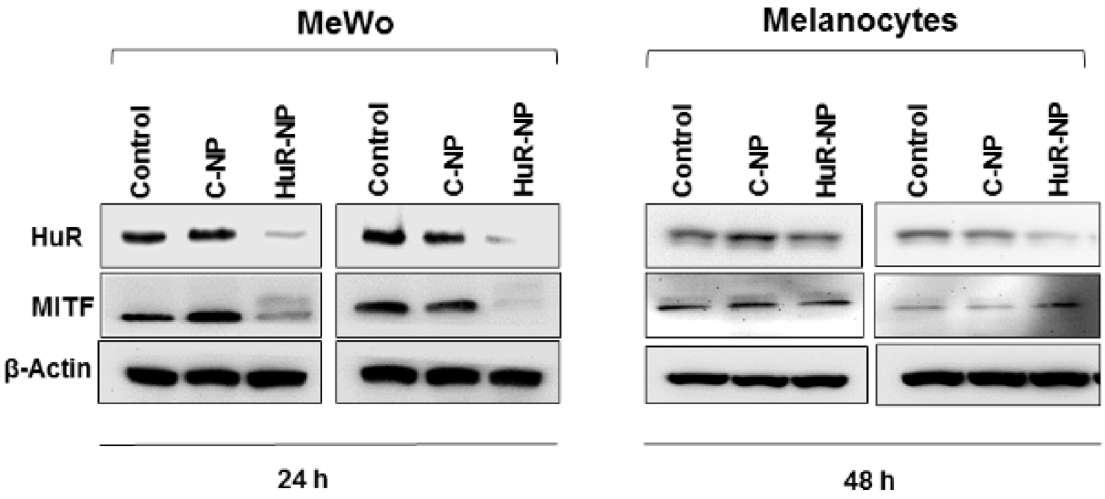

Figure S7. HuR-NP treatment reduced MITF in melanoma (MeWo) cell line but not in melanocytes at both 24 h and 48 h after treatment. No change in MITF expression was observed in C-NP-treated and

untreated control cells in both, MeWo cell line and melanocytes. Beta-actin was used as a loading control.

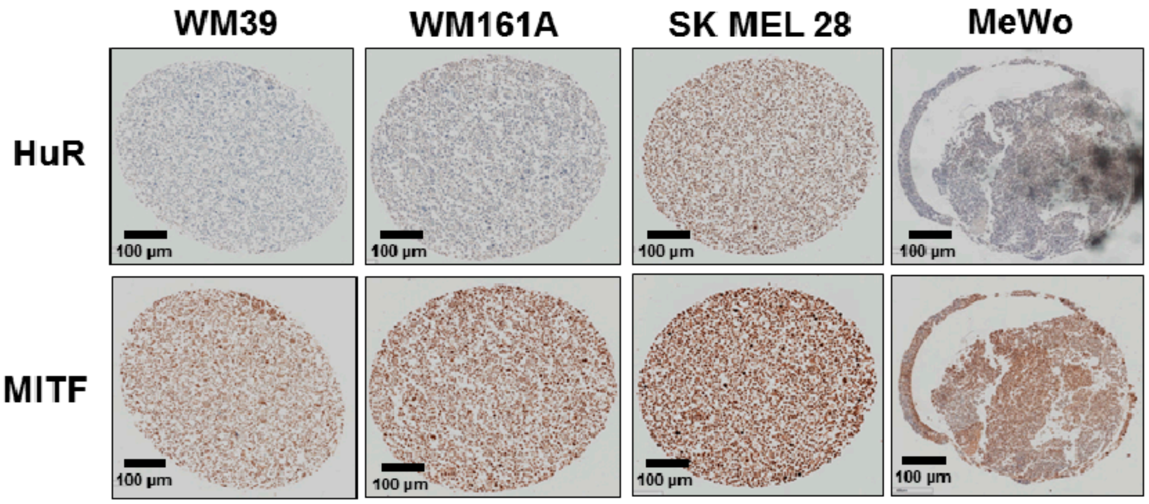

Figure S8. Immunocytochemistry of HuR and MITF expression in a panel of human melanoma cell lines. Scale bar, 100 µm.

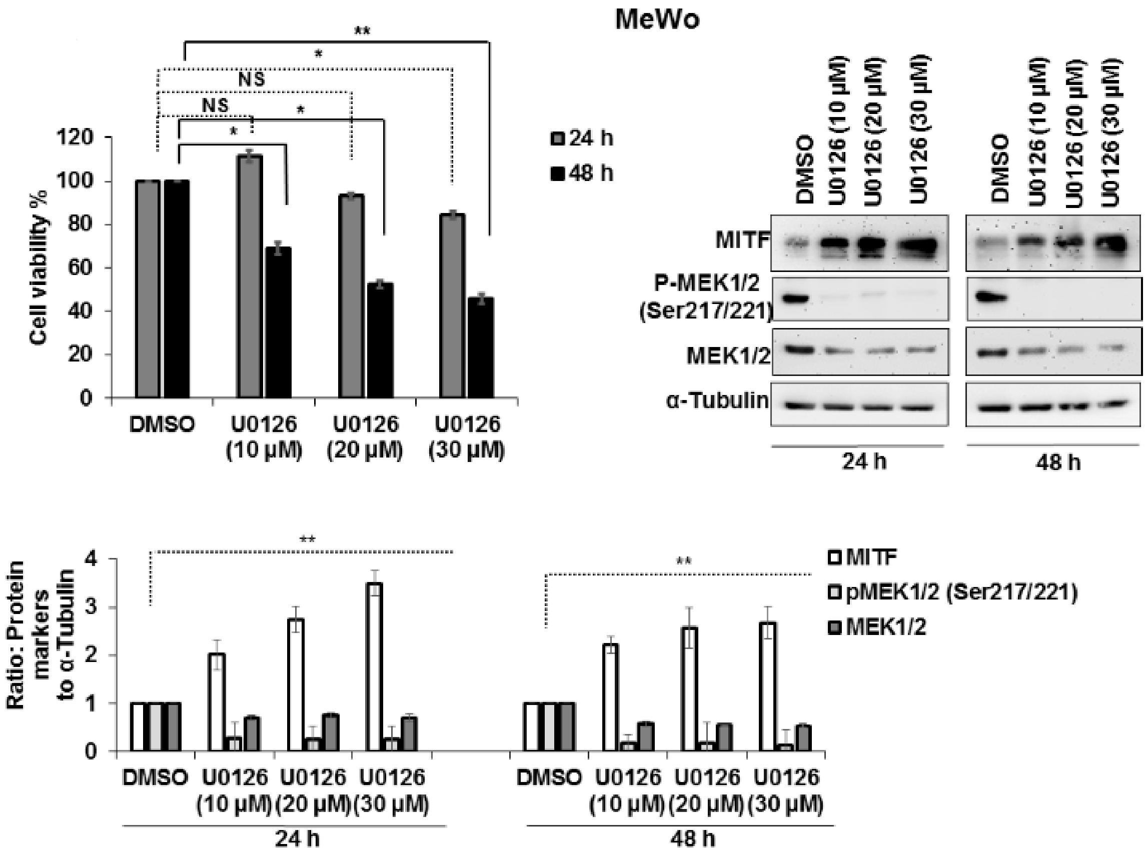

Figure S9. Cytotoxic effect of U0126 on MeWo melanoma cell line. Cells were treated with 10 µM, 20 µM and 30 µM of U0126 and assessed for cell viability by trypan blue exclusion assay, and for protein expression by western blotting. DMSO treated cells served as controls. U0126 produced a concentration-dependent reduction in cell viability. Western blotting showed U0126 reduced P-MEK1/2(Ser217/221) and total MEK1/2 expression while concomitantly increasing MITF expression. α-Tubulin was used as an internal loading control. Bar graphs represent semi-quantitative analysis of the protein expression in U0126 treated MeWo cells. Error bar denotes SD; NS not significant; *$p < 0.05$; **$p < 0.01$.

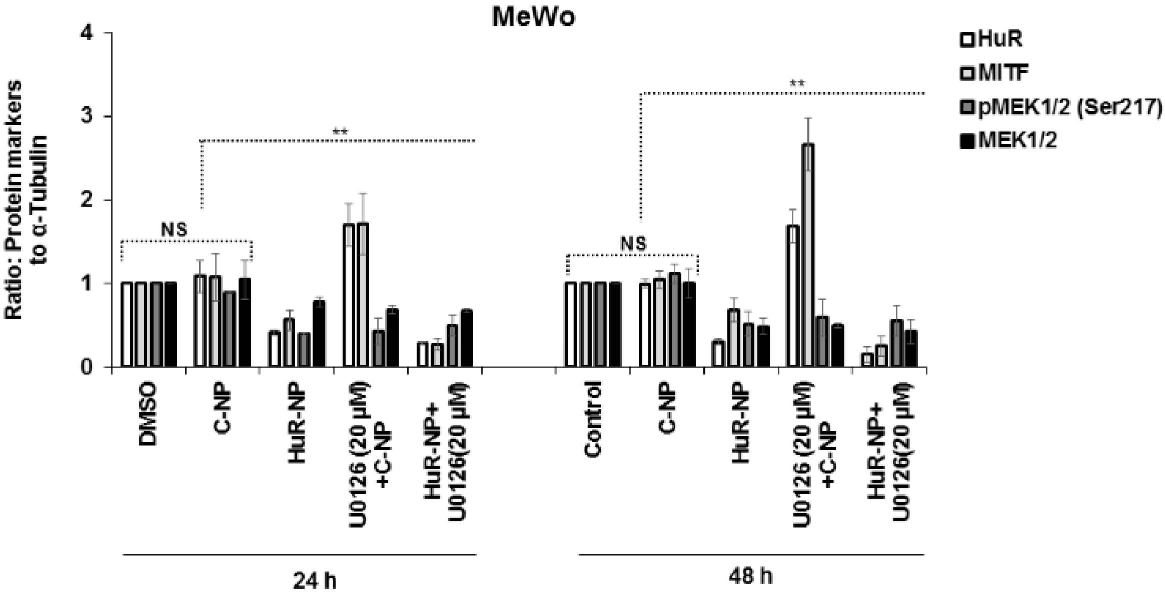

Figure S10. Bar graphs represent semi-quantitative analysis of the protein expression detected by western blot analysis in MeWo melanoma cells treated with C-NP, HuR-NP, U0126 and combination of C-NP or HuR-NP with U0126 at 24 h and 48 h after treatment. DMSO treated cells served as control. Error bar denotes SD; **p < 0.01.

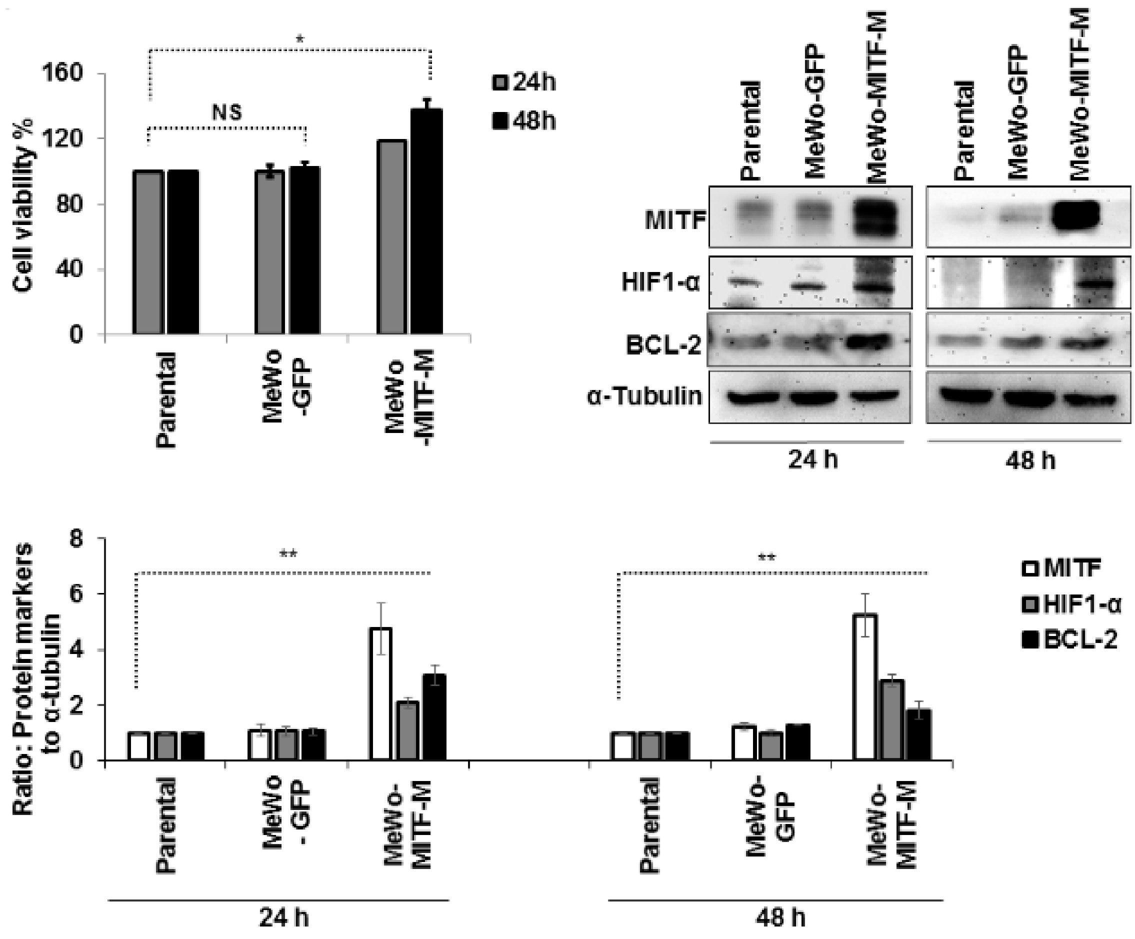

Figure S11. Cell viability of MeWo-MITF-M compared to MeWo-GFP and parental MeWo cells were assessed by trypan blue exclusion assay at 24 h and 48 h. MITF, HIF1-*α* and BCL-2 protein expression

were increased in MeWo-MITF cells compared MeWo-GFP cells. α-Tubulin was used as an internal loading control. Bar graphs represent semi-quantitative analysis of MITF, HIF1-α and BCL-2 expression at 24 h and 48 h after treatment. Error bar denotes SD; *$p < 0.05$; **$p < 0.01$.

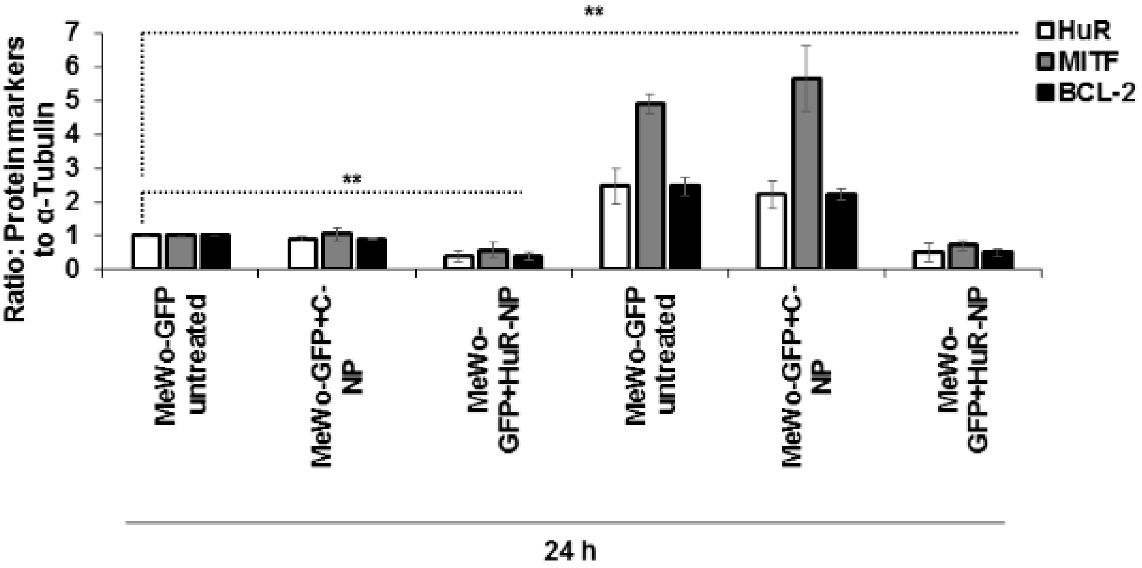

Figure S12. Semi-quantitative analysis of the protein expression in C-NP and HuR-NP treated MeWo-GFP and MeWo-MITF-M cells detected by western blot at 24 h after treatment. Error bar denotes SD; **$p < 0.001$.

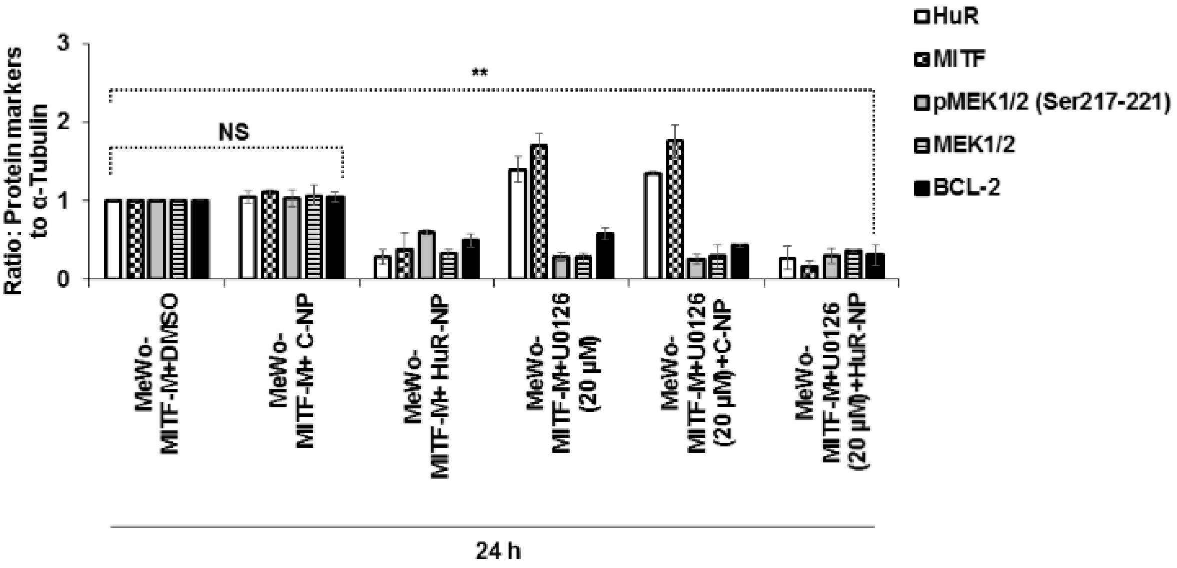

Figure S13. Bar graphs represent semi-quantitative analysis of the protein expression detected in MeWo-MITF-M cells treated with C-NP, HuR-NP, U0126 (20 μM) and combination of U0126 with C-NP or HuR-NP by western blot at 24 h post-treatment. DMSO treated MeWo-MITF-M cells served as control. Error bar denotes SD; **$p < 0.01$.

Original figures about the western blot

Figure. 2

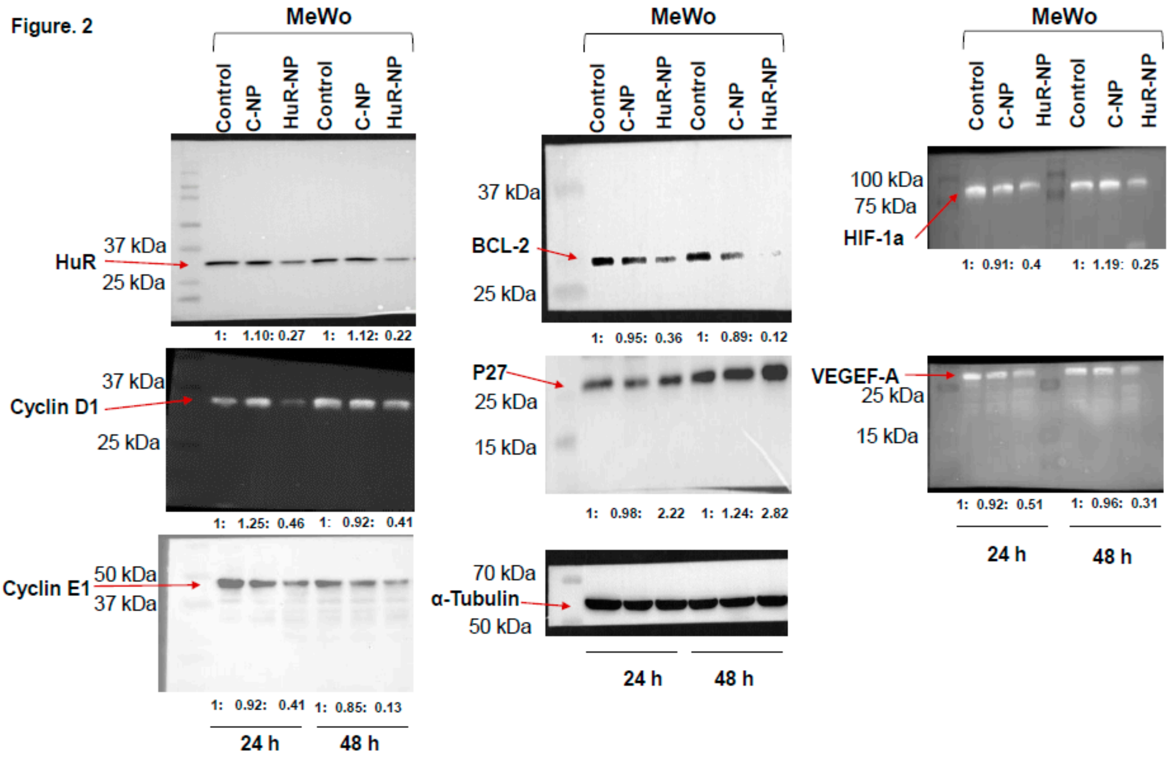

Figure. 2 continue

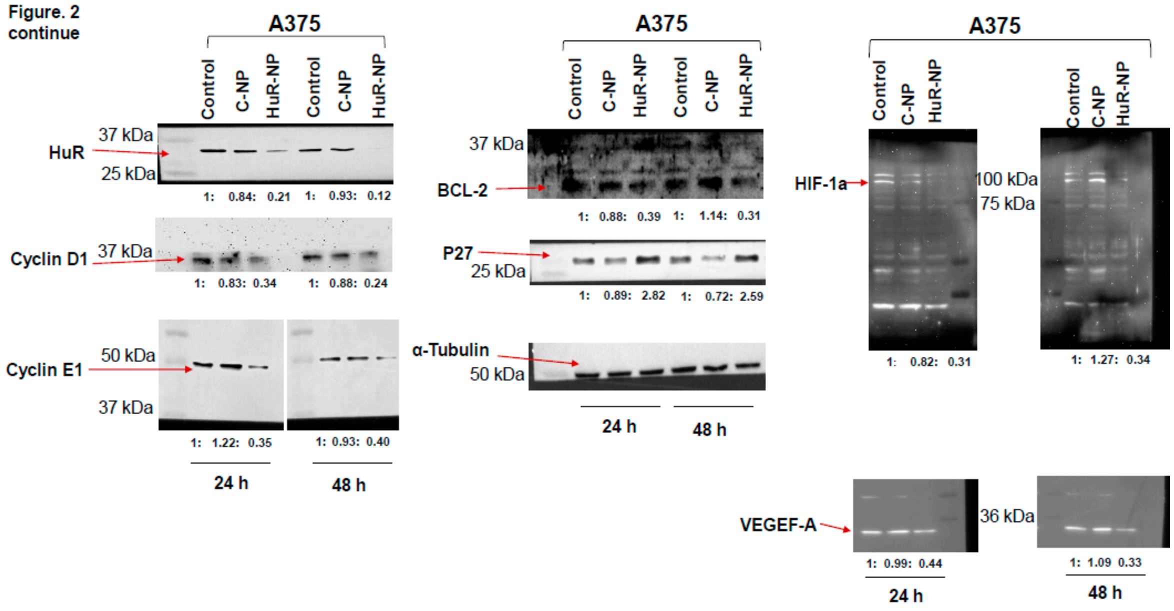

Figure. 2 continue

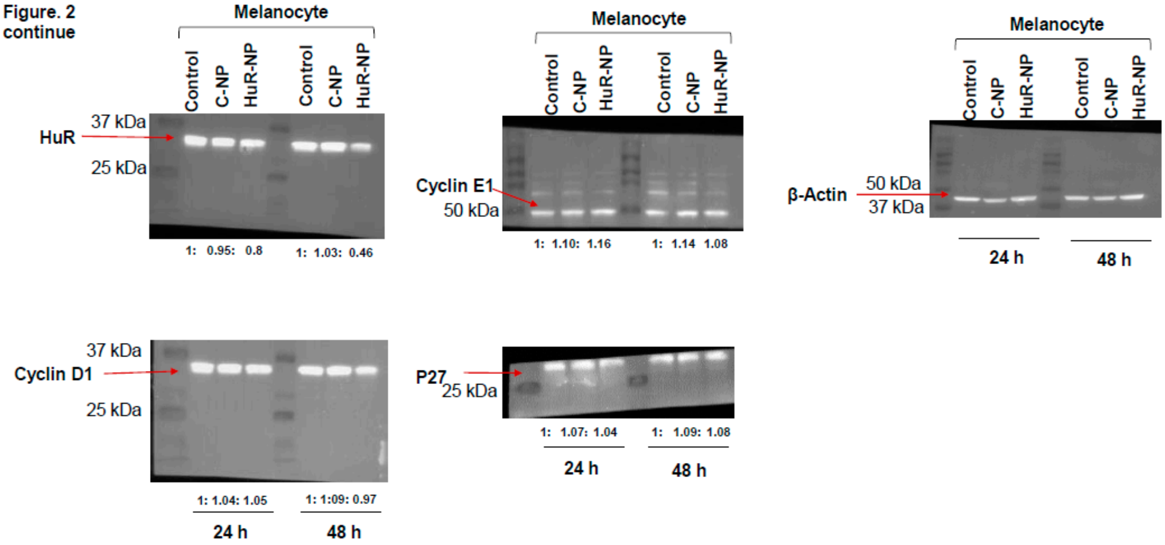

Figure. 4 (A)

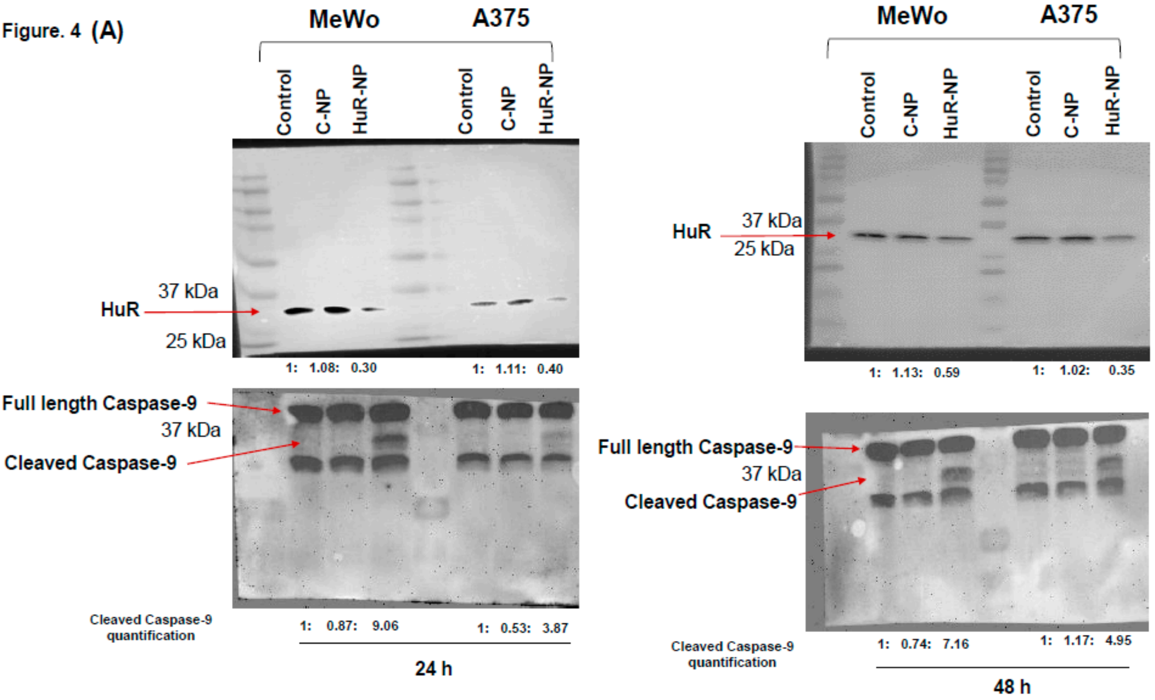

Figure. 4 (A) continue

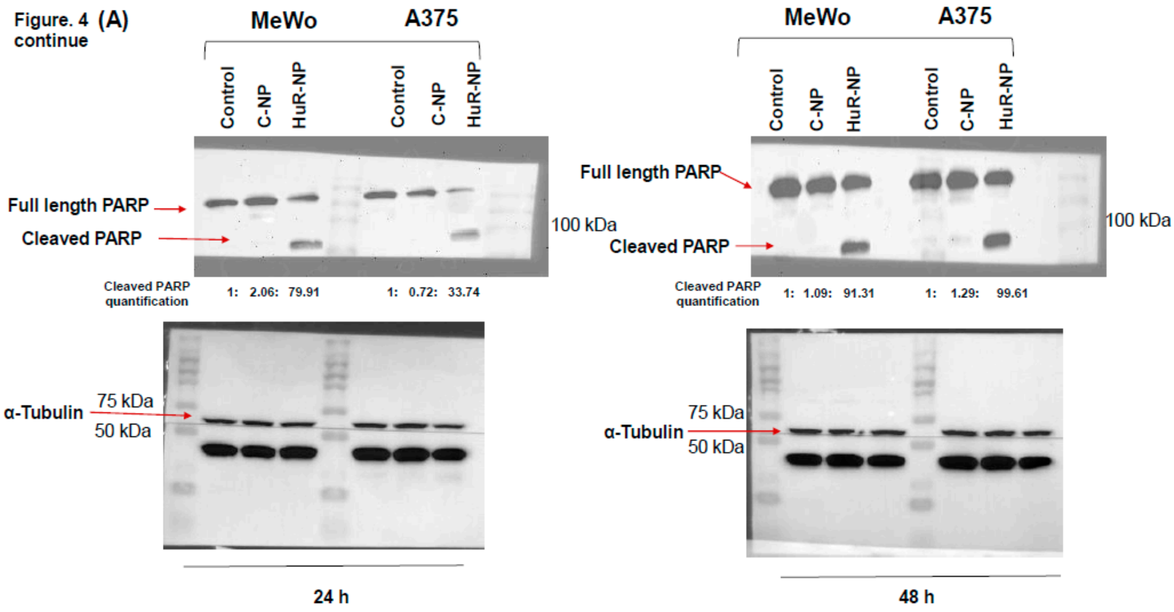

Figure. 6 (C)

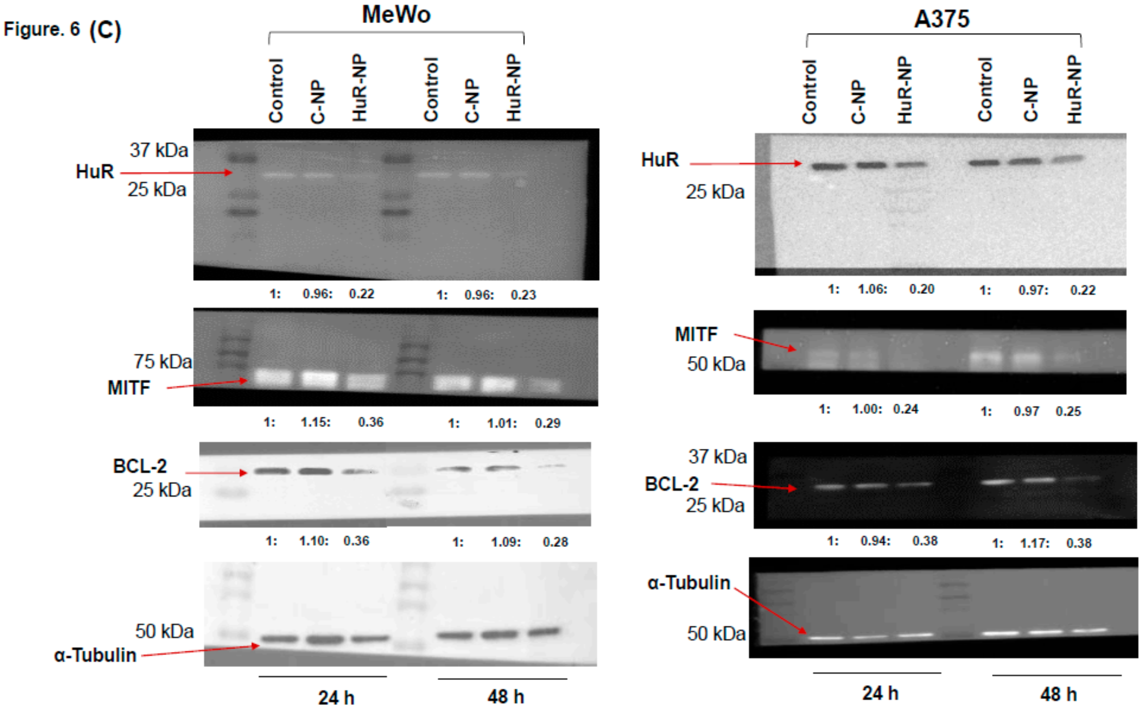

Figure. 7

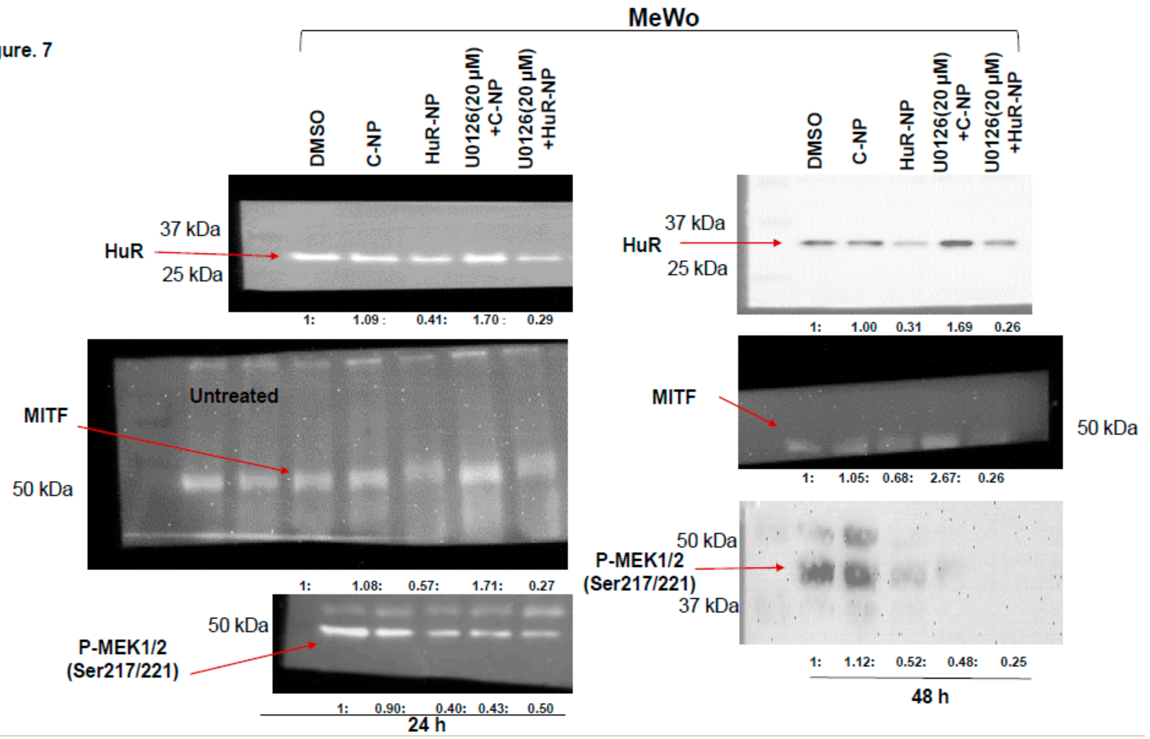

Figure. 7
continue

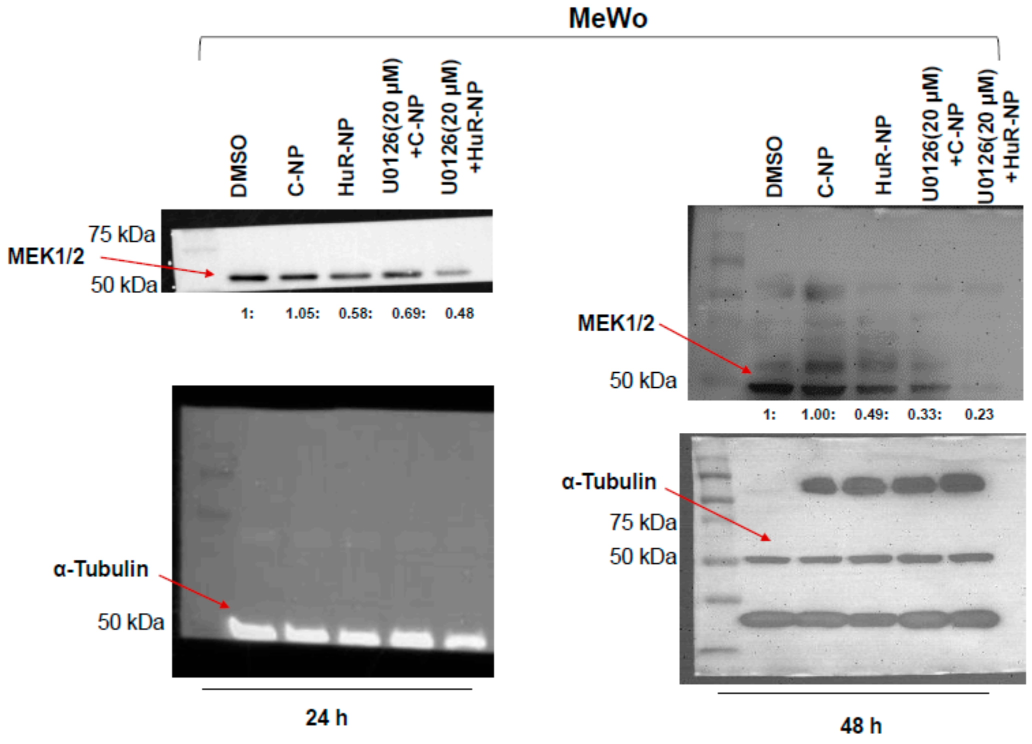

Figure. 8

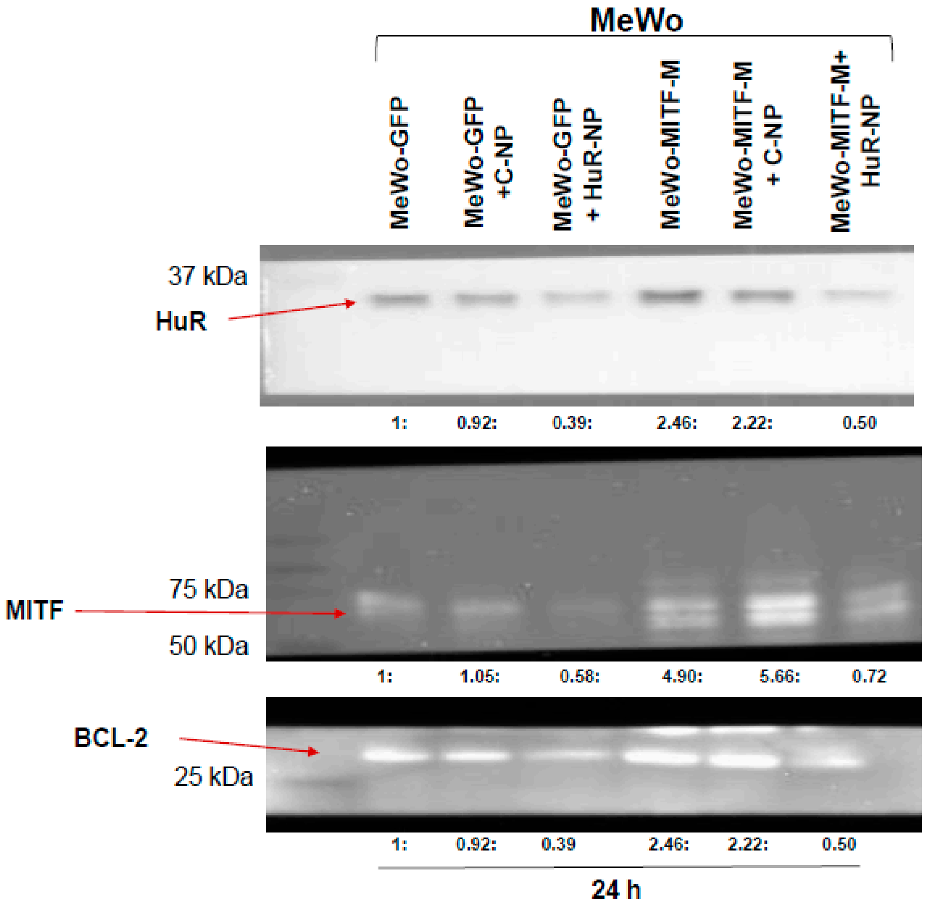

Figure. 8
continue

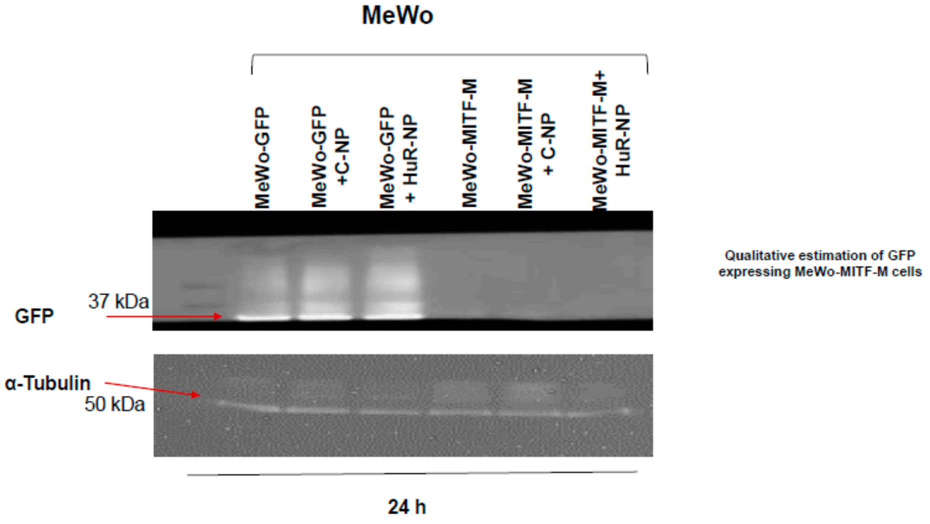

Figure. 9

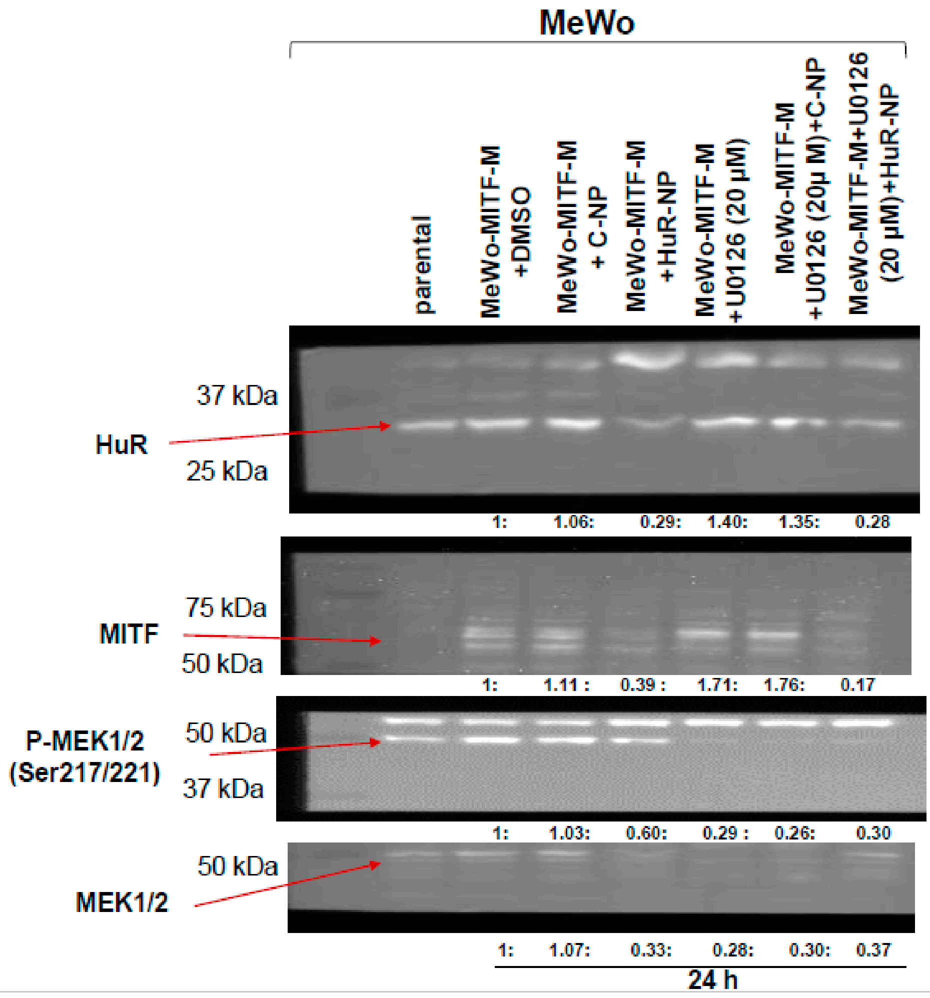

Figure. 9
continue

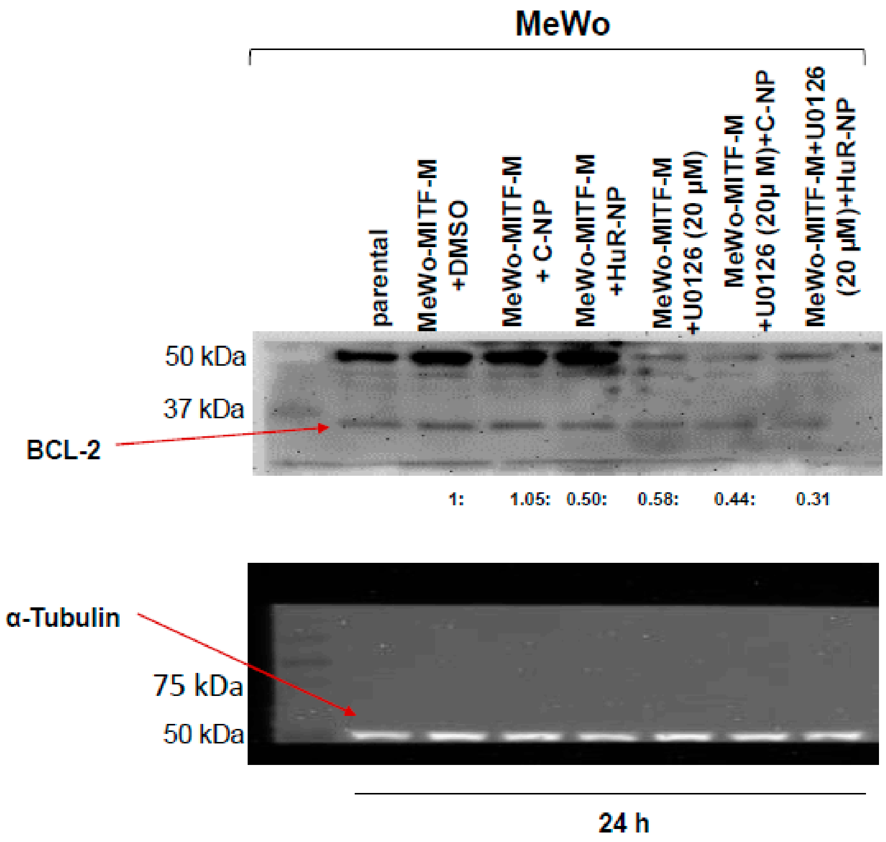

Supplementary Figure. 1

Qualitative baseline estimation

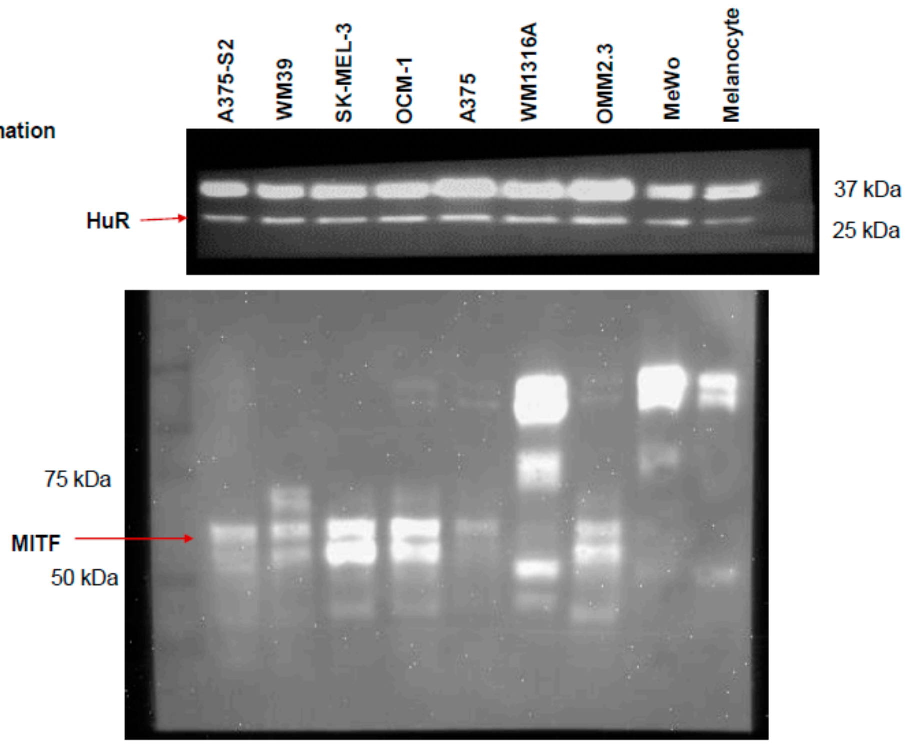

Supplementary Figure. 1
Continue

Qualitative baseline estimation

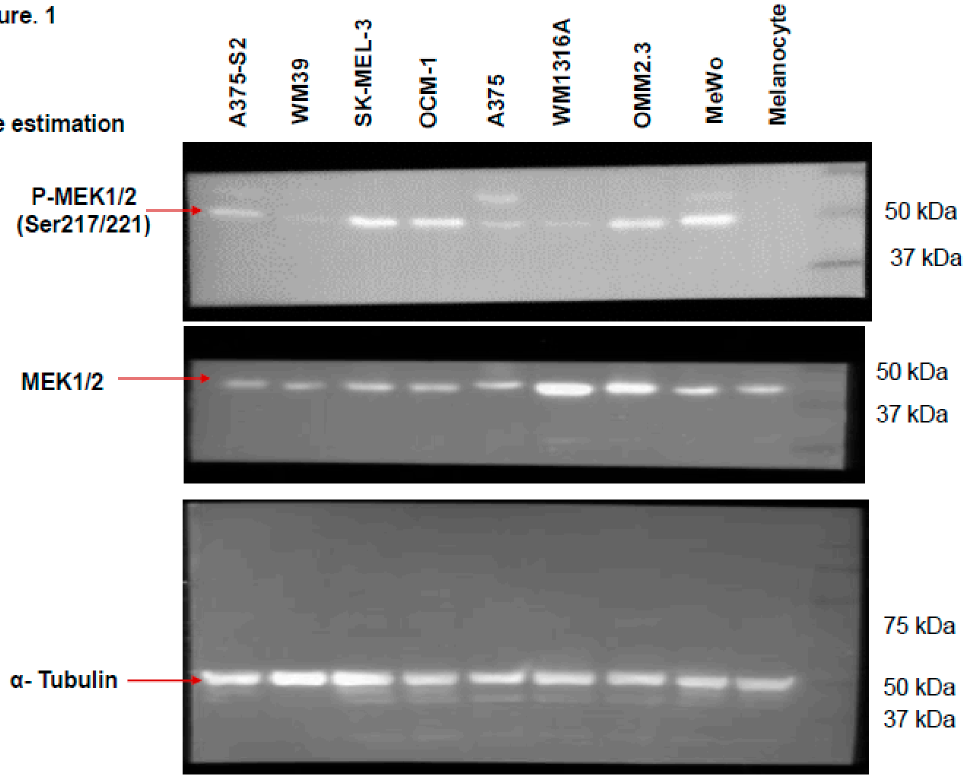

Supplementary Figure. 2

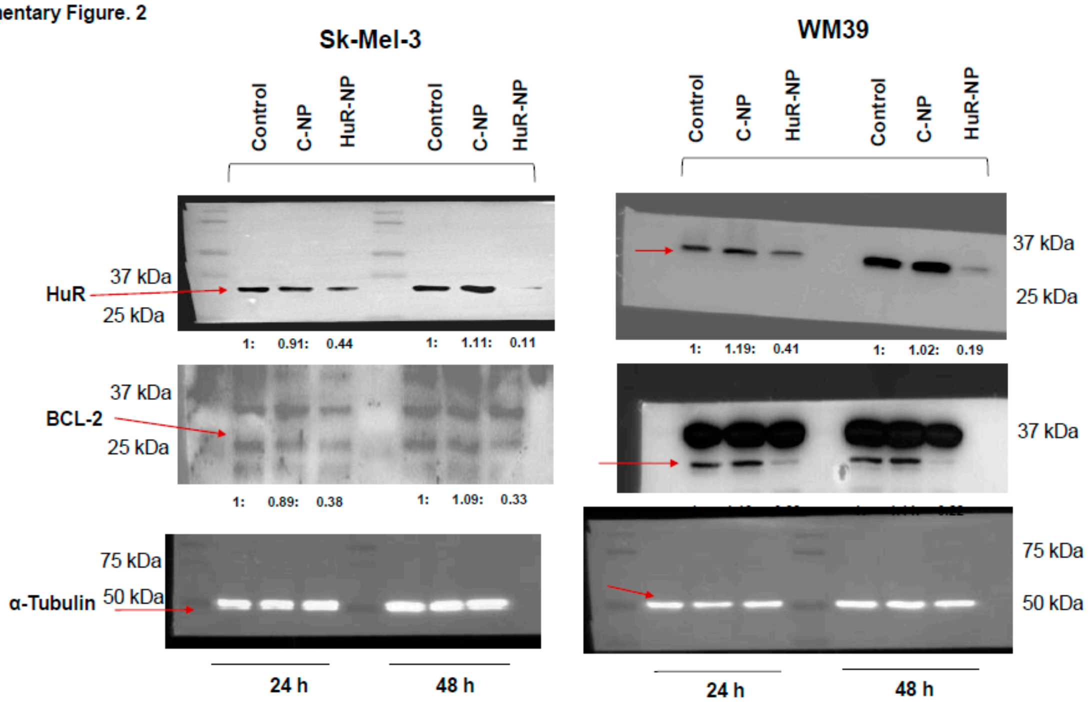

Supplementary Figure. 5

(A)

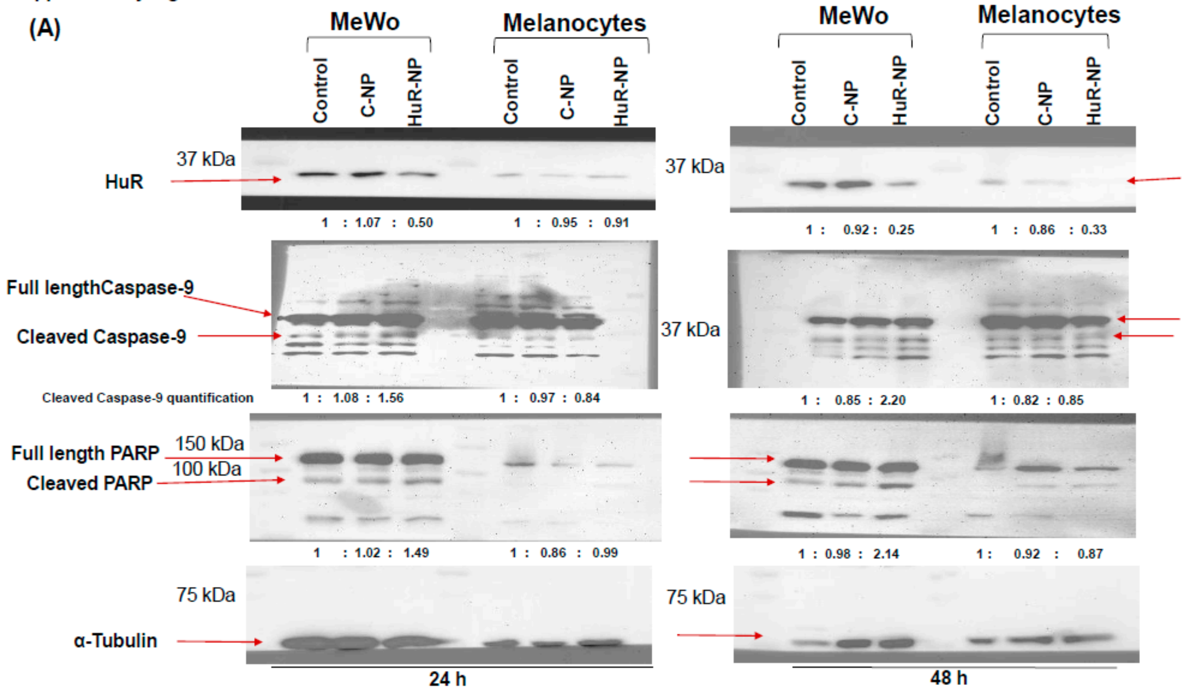

Supplementary Figure. 7

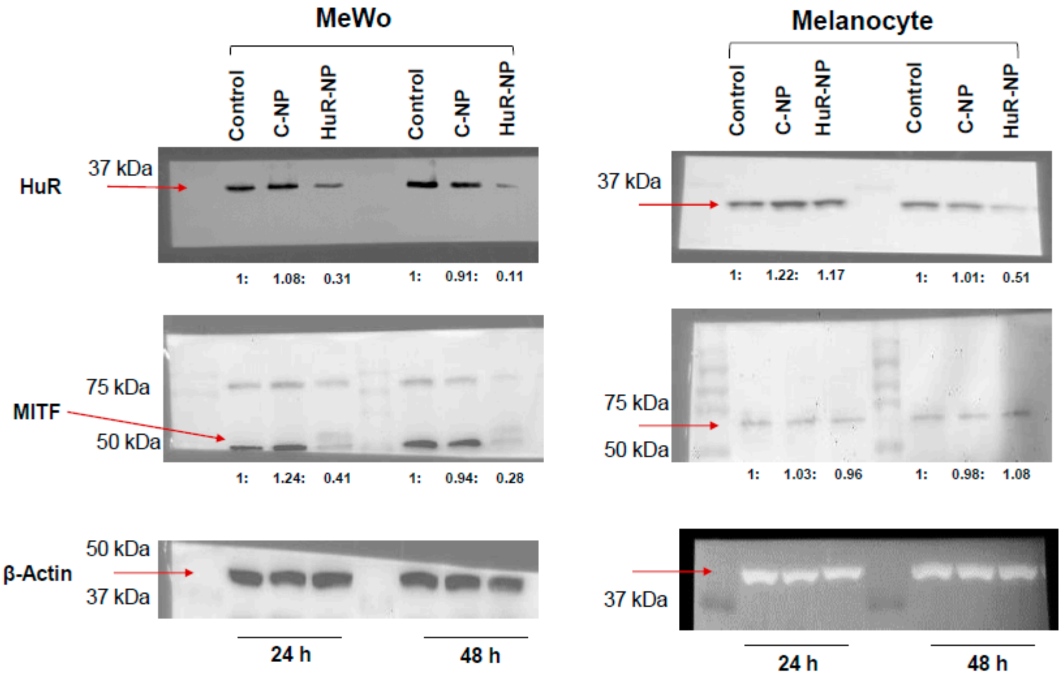

Supplementary Figure. 9

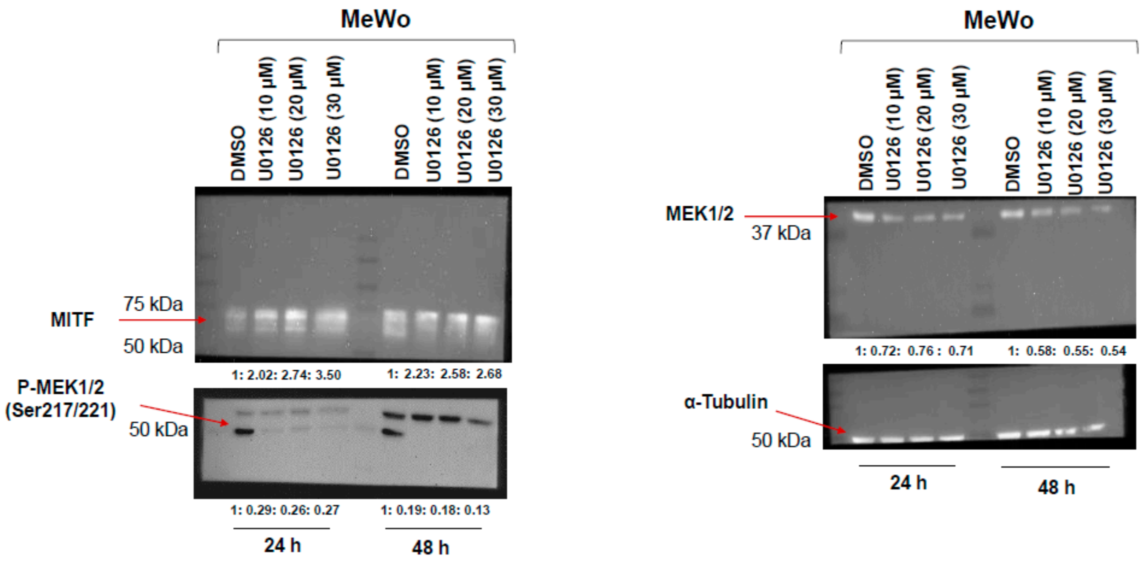

Supplementary Figure. 11

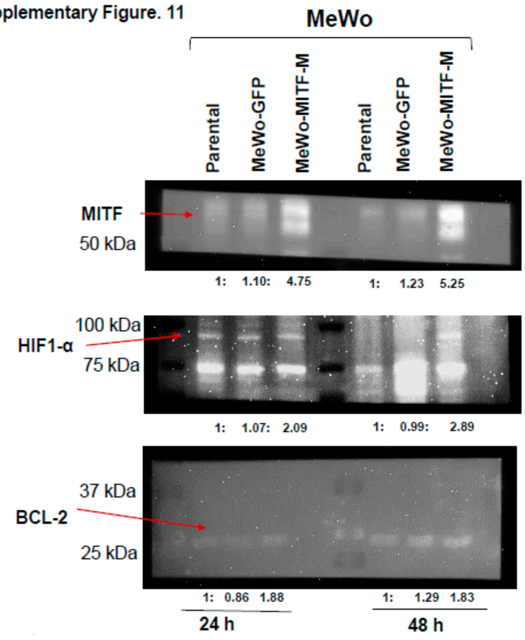

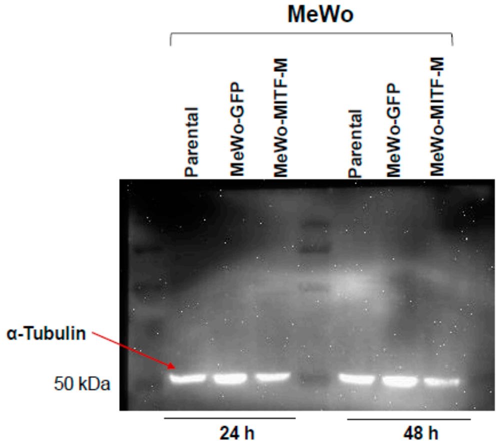

Supplement: Supplementary file 1 [file cancers-13-00166-s001.pdf]
